# Circadian and environmental signal integration in a natural population of *Arabidopsis*

**DOI:** 10.1073/pnas.2402697121

**Published:** 2024-08-22

**Authors:** Haruki Nishio, Dora L. Cano-Ramirez, Tomoaki Muranaka, Luíza Lane de Barros Dantas, Mie N. Honjo, Jiro Sugisaka, Hiroshi Kudoh, Antony N. Dodd

**Affiliations:** ^a^Center for Ecological Research, Kyoto University, Otsu, Shiga 520-2113, Japan; ^b^Data Science and AI Innovation Research Promotion Center, Shiga University, Hikone, Shiga 522-8522, Japan; ^c^The Sainsbury Laboratory, University of Cambridge, Cambridge CB2 1LR, United Kingdom; ^d^School of Biological Sciences, University of Bristol, Bristol BS8 1TQ, United Kingdom; ^e^Graduate School of Bioagricultural Sciences, Nagoya University, Nagoya, Aichi 464-0814, Japan; ^f^Department of Cell and Developmental Biology, John Innes Centre, Norwich NR4 7RU, United Kingdom

**Keywords:** circadian rhythms, signal transduction, field biology, state-space modeling

## Abstract

Plants sense and respond to environmental cues during 24 h fluctuations in their environment. This requires the integration of internal cues such as circadian timing with environmental cues such as light and temperature to elicit cellular responses through signal transduction. However, the integration and transduction of circadian and environmental signals by plants growing in natural environments remains poorly understood. To gain insights into 24 h dynamics of environmental signaling in nature, we performed a field study of signal transduction from the nucleus to chloroplasts in a natural population of *Arabidopsis halleri.* Using several modeling approaches to interpret the data, we identified that the circadian clock and temperature are key regulators of this pathway under natural conditions. We identified potential time-delay steps between pathway components, and diel fluctuations in the response of the pathway to temperature cues that are reminiscent of the process of circadian gating. We found that our modeling framework can be extended to other signaling pathways that undergo diel oscillations and respond to environmental cues. This approach of combining studies of gene expression in the field with modeling allowed us to identify the dynamic integration and transduction of environmental cues, in plant cells, under naturally fluctuating diel cycles.

## Significance

A major question in circadian biology is of how the molecular processes associated with circadian clocks operate in a natural setting. Using a natural population of *Arabidopsis* plants as an experimental model, we investigated how circadian timing signals are combined with information about the ambient light and temperature conditions, to regulate an intracellular signal transduction pathway. We identified multiple locations of environmental input within the signaling pathway, mathematically inferred causality within the pathway, and revealed 24 h fluctuations in environmental sensitivity, under field conditions, that are reminiscent of the process of circadian gating. Our approaches are of universal value for investigating circadian-regulated processes under natural conditions.

Plants have sophisticated environmental sensing and signaling mechanisms that underpin their responses to the fluctuating environment. Under natural conditions, signaling pathways that integrate dynamic, overlapping, and complex environmental stimuli are required (1, 2). These environmental fluctuations include an oscillating component with a 24 h period, which arises from the cycle of day and night. Circadian clocks, which are endogenous biological oscillators, provide a cellular estimate of the time of day that coordinates the diel responses of plants with environmental fluctuations by aligning transcription, metabolism, and development with the time of day (3–8). In plants, environmental information including the light and temperature conditions is used to adjust the phase of the circadian oscillator, through the process of entrainment, so that the phase is aligned with the 24 h environmental cycle. This alignment between the circadian oscillator and the 24 h environmental cycle contributes to the fitness of plants (5).

Under natural conditions, environmental cues are a major determinant of temporal programs of gene expression (9). For example, 97% of diel transcript profiles in field-grown rice can be predicted from meteorological data (9), and temperature cues regulate the alternative splicing of transcripts encoding circadian oscillator components in field-grown sugarcane (10). Recent studies have provided insights into the diel organization of the transcriptome and metabolism, under field conditions, for several crops and *Arabidopsis* species (9–18). A question that arises from these studies is how information from the circadian clock and environmental cues are processed to form integrated outputs. The diel dynamics of environmental signaling pathways, with defined inputs and outputs, are less well understood under natural conditions. Their behavior under controlled laboratory conditions do not necessarily reflect the dynamics in the field, even when using sophisticated replication of field conditions (15, 17). Therefore, analyzing the dynamics of cellular processes within complex fluctuating natural conditions allows us to elucidate how molecular mechanisms identified under controlled laboratory conditions operate in a natural setting (15). Understanding this is valuable for translating laboratory studies into crop improvement. Furthermore, because plants represent the major part of organismal biomass on Earth, it could contribute to forecasting ecosystem responses to increasingly unpredictable climates (19, 20).

**Fig. 1.**
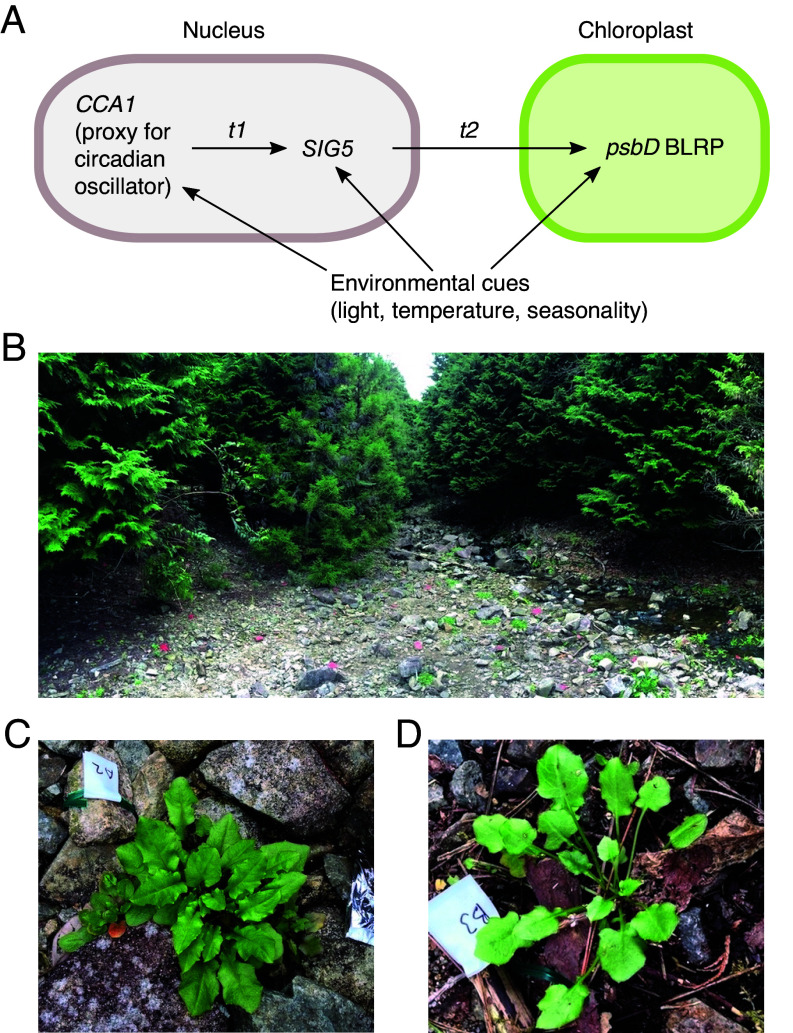
Circadian and environmental signal integration in a natural population of *A. halleri*. (*A*) Potential architecture of a signal transduction pathway underlying SIG5-mediated signaling to chloroplasts, with environmental inputs occurring at several positions. *t1* and *t2* represent the time taken for signal transduction between each pathway component. (*B*) A photograph of the Omoide-gawa river site, which has naturally occurring populations of *A. halleri*. The majority of plants at the ground level on the stony riverbanks are *A. halleri*. (*C* and *D*) Examples of rosette-stage *A. halleri* plants growing under (*C*) sun and (*D*) shade conditions. All photographs were taken during the September 2015 sampling season.

To study the integration and transduction of circadian and environmental signals under naturally fluctuating conditions, we selected a well-characterized environmental signaling pathway as a model system. This model comprises the regulation of three components; a nuclear-encoded key component of the *Arabidopsis* circadian clock (*CIRCADIAN CLOCK ASSOCIATED 1*, *CCA1*), a nuclear-encoded regulator of chloroplast transcription (*SIGMA FACTOR 5*, *SIG5*), and the chloroplast-encoded gene *psbD*, which encodes the D2 protein of Photosystem II (21–23) (Fig. 1*A*). *CCA1* transcript abundance can be used as a proxy for the status of the circadian clock. *SIG5* is regulated closely by the circadian oscillator under constant conditions (22), and its transcript abundance responds to light, temperature, and other environmental cues (21–33), which made us reason that SIG5 is an integrator of circadian and environmental information. SIG5 is imported into chloroplasts, and regulates transcription from the blue light responsive promoter of *psbD* (*psbD* BLRP) (21) (Fig. 1*A*). Thus, we assumed that SIG5-mediated signaling to chloroplasts involves a hierarchically organized pathway, whereby *CCA1* is positioned upstream from the regulation of *SIG5* transcript accumulation, and *psbD* BLRP is positioned downstream of *SIG5* activity (Fig. 1*A*). We also assumed that environmental signals might influence each component independently (Fig. 1*A*) (34). We chose this pathway because it includes three major components representing distinct regulatory points of signal transduction, and has a relatively low level of complexity to evaluate circadian and environmental signal integration and transduction under realistic field conditions.

By targeting this model pathway, we combined time-series gene expression data with statistical modeling to investigate the temporal dynamics of signal integration and transduction in a fluctuating natural environment. We conducted the study using a natural population of a perennial *Arabidopsis* (*A. halleri* subsp. *gemmifera*, referred to as *A. halleri*) (Fig. 1 *B*–*D*) (35). The close-relatedness of *A. halleri* and *Arabidopsis thaliana* makes it possible to identify pairs of homologous genes based on the sequence similarity (indicated by *Ahg* or *At* prefixes to gene names) (36). The circadian clock-SIG5-*psbD* BLRP pathway is present in *A. thaliana* and *A. halleri*, and well-conserved across the vascular plants (37, 38). We obtained several time series, during two seasons of the year, that monitored pathway function under representative light and temperature conditions in *A. halleri* in its natural habitat.

Interpreting time-series transcript data derived from complex fluctuating environments is challenging. We used statistical models, rather than models of biochemical kinetics (39), because this provides an effective tool for interpreting the influences of external factors on the response variables. We applied state-space modeling that integrates observations and the latent true state of a dynamic system into a statistical model. It is often used to interpret trends and oscillations in a system, and to evaluate the effects of external drivers on time-series data (40–42). It explicitly formulates the system noise and observation error as state and observation equations, respectively. By incorporating an autoregressive trend and exogenous influences into a state space model, we estimated the dynamics of each pathway component that arises from its internal dynamics and external factors. Comparable approaches have allowed the investigation of diel and seasonal changes of transcriptome dynamics in *A. halleri* (12, 43, 44) and rice (9).

We evaluated evidence for causal regulation between the pathway components using convergent cross-mapping (CCM), which provides a quantitative test for causation in time-series of coupled dynamical systems (45). In contrast to our predictive state-space models, CCM evaluates whether the historical fluctuation in one variable can be used to estimate the fluctuation in a second variable (45). This has been used previously to investigate a variety of ecological processes, including the seasonal regulation of gene expression by epigenetic marks in a natural *A. halleri* population (44) and the seasonal transcriptome in Japanese beech (46). Using these approaches, we identified key roles for temperature and the circadian clock in the regulation of this signaling pathway under natural conditions. Our approaches could be applicable to the study of many circadian-regulated processes under naturally fluctuating conditions.

## Results

### Biological Data Underlying Models of Signal Transduction.

**Fig. 2.**
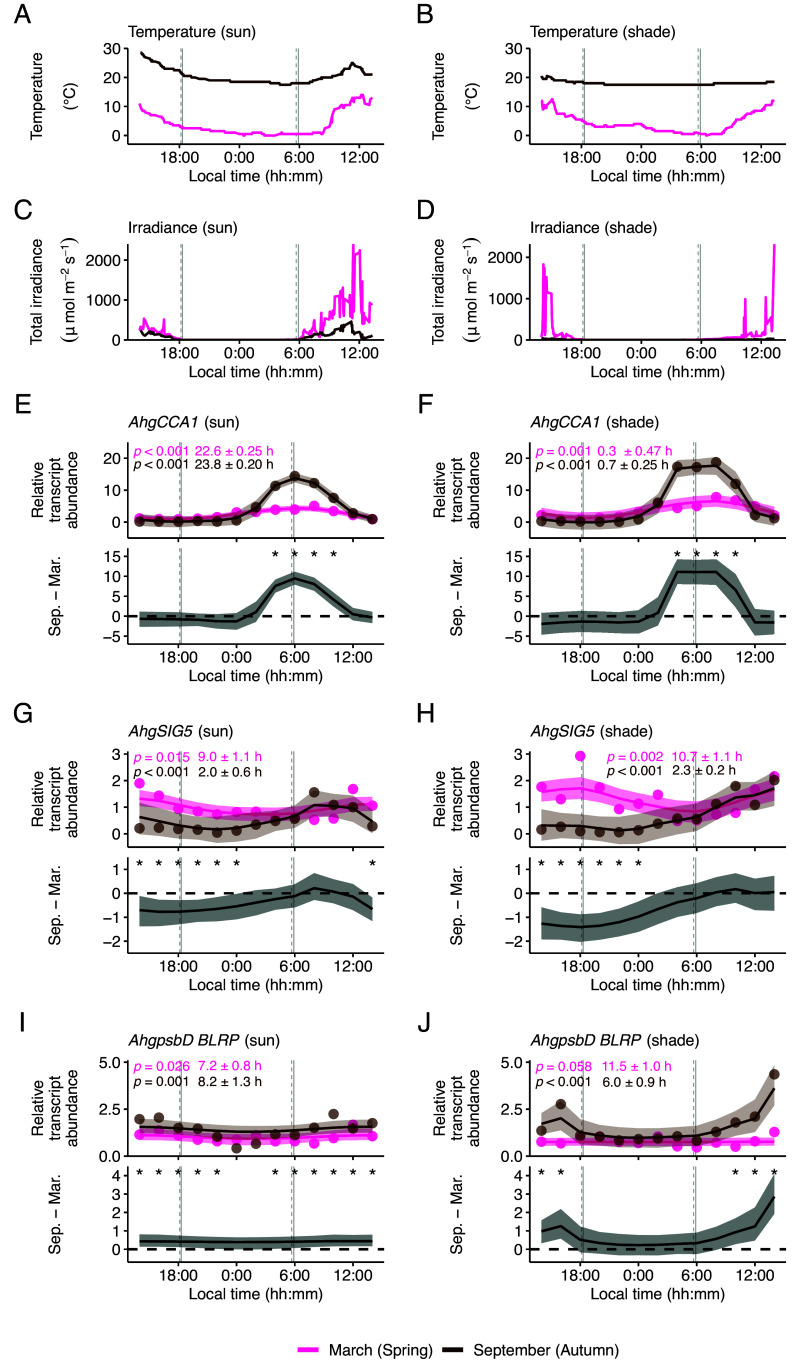
Distinct diel dynamics of components of a circadian signaling pathway between spring and autumn in a natural population of *A. halleri*. (*A*–*D*) Diel fluctuations in (*A* and *B*) ambient temperature and (*C* and *D*) total irradiance (200 to 900 nm), measured at 5-min intervals during the sampling period in March and September 2015. (*E*–*J*) Bayesian estimation of STM for transcript dynamics of (*E* and *F*) *AhgCCA1*, (*G* and *H*) *AhgSIG5* and (*I* and *J*) *AhgpsbD BLRP*. In each panel, the upper graphs show the predicted relative transcript abundance for March (pink; $\mu_{1}$, equation 1 in *SI Appendix*, *Supporting Text*) and September (brown; $\mu_{2}$, equation 3) with the mean of observed values (dots), and the lower graphs represent the differences in transcript abundance between March and September (δ, equation 2). The solid line and the shaded region are the median and the 95% CI of the posterior distribution. When the 95% CI of the difference between March and September does not contain zero, the difference is considered significant and is indicated by asterisks. Panels *E*–*J* include estimation of rhythmicity and peak time relative to solar dawn of underlying data, using JTK_CYCLE. Vertical gray lines on time-series plots indicate the times of sunrise and sunset during March (solid line) and September (dashed). STM analysis used data from six replicate plants per condition.

Under controlled conditions of constant light, *AtCCA1* and *AtSIG5* transcript abundance are well correlated (*SI Appendix*, Fig. S1 *A*–*D*; data from refs. 3, 4, 6, 22, and 47). This correlation between *AtCCA1* and *AtSIG5* transcript abundance is absent under light/dark cycles (*SI Appendix*, Fig. S1 *E* and *F*; data from refs. 22 and 48), suggesting that the integration of light and dark cues alters the diel regulation of *AtSIG5* transcript accumulation. To investigate these processes of signal integration under natural conditions, we acquired time-series of transcript abundance during spring (March) and autumn/fall (September), close to the spring or autumn equinox (*SI Appendix*, Figs. S2 and S3). Although both the spring and autumn equinoxes share 12 h photoperiods, they provide contrasting temperature regimes (cool and warm, respectively) (Fig. 2 *A* and *B*), with irradiance levels determined by weather conditions (Fig. 2 *C* and *D*). This allowed us to investigate temperature, light and, seasonal influences upon SIG5-mediated signaling to chloroplasts, because this pathway is known to be affected by light and temperature in *A. thaliana* (21, 23, 24, 28, 33, 37). We obtained data from areas with open sky and with vegetational shade, to include within our models the transcriptional responses to a wider range of irradiance levels (Fig. 2 *C* and *D*). The “sun” and “shade” sampling sites were chosen by measurement of the ratio of red to far-red light (R:FR) (*SI Appendix*, Fig. S4) and availability of plant patches, because *A. halleri* does not grow in deep shade. The total light intensity at the sun sampling site was 5- to 10-fold greater during March 2015 than during September 2015, depending on the time of day, due to weather differences (Fig. 2 *C* and *D*). During March 2015, the study site temperature ranged from 0 °C to 14 °C at the sun site, and from 0 °C to 13 °C at the shade site (Fig. 2 *A* and *B*). The temperature was always above 17 °C during September 2015, with greater diel fluctuations at the sun site (Fig. 2 *A* and *B*).

We monitored a 24 h cycle of abundance of *AhgCCA1*, *AhgSIG5,* and *AhgpsbD* BLRP transcripts under each of these conditions, using RT-qPCR (*SI Appendix*, Figs. S2 and S3). Data from all the experiments were normalized to a single reference sample collected at midday, to enable comparability between sampling seasons and conditions. *AhgCCA1* oscillated with a 24 h cycle, reaching peak abundance at or just after astronomical dawn (*SI Appendix*, Fig. S2 *E* and *F*), whereas *AhgSIG5* and *AhgpsbD* BLRP reached peak abundance between the middle and end of the photoperiod, depending on conditions (*SI Appendix*, Fig. S2 *G*–*J*). The magnitude (diel fold-change) of oscillation of each transcript varied according to the sampling season and light conditions, with all transcripts being rhythmic apart from *AhgpsbD* BLRP under shade conditions, during the March sampling (*SI Appendix*, Fig. S2 *E*–*J*). *AhgCCA1* transcripts underwent a fivefold (sun) and sevenfold (shade) change over the 24 h cycle during the March sampling, compared with a 98-fold (sun) and 190-fold (shade) change during the September sampling (fold-changes based on peak and trough abundance). This greater magnitude of oscillation occurred also for *AhgSIG5* during September (sun 38-fold; shade 33-fold) compared with March (sun 2.7-fold, shade 5.0-fold), and also for *AhgpsbD* BLRP during September (4.3-fold in sun and shade conditions) compared with March (sun 1.7-fold, shade 1.3-fold). Therefore, the relative magnitude of *AhgCCA1* transcript oscillation aligned well with that of *AhgSIG5* and *AhgpsbD* BLRP, whereby stronger diel cycling occurred in September than in March. Furthermore, the magnitude (fold-change) of the 24 h oscillation of *AhgCCA1* was always greater than for *AhgSIG5* and *AhgpsbD* BLRP under any given condition.

We compared the pathway dynamics between the spring and autumn sampling periods by using a smooth trend model (referred to here as STM). This is a type of state-space modeling that allows inference of a trend over time, where there is sampling noise and environmental stochasticity (49). We estimated the parameters of a STM by Bayesian inference using the Markov Chain Monte Carlo (MCMC) approach (42, 50) to visualize the differences in transcript abundance between the spring and autumn sampling periods (convergence of MCMC sampling is shown in *SI Appendix*, Figs. S5–S7). The STM reproduced well the dynamics of the observed transcript levels [Pearson’s correlation coefficient = 1.00 for *AhgCCA1* (sun), 0.99 for *AhgCCA1* (shade), 0.89 for *AhgSIG5* (sun), 0.90 for *AhgSIG5* (shade), 0.67 for *AhgpsbD* BLRP (sun), and 0.95 for *AhgpsbD* BLRP (shade)]. To further evaluate the model fit, we performed a residual analysis (*SI Appendix*, Fig. S8). Residuals against the fitted values showed no specific trend around zero, supporting the validity of the model (*SI Appendix*, Fig. S8 *A*, *C*, *E*, *G*, *I*, and *K*). In support of the model, quantile–quantile plots of the residuals showed that they generally follow normal distributions, with the exception of *AhgCCA1* (sun), *AhgSIG5* (sun), and *AhgpsbD* BLRP (shade) showing deviations from the theoretical lines at higher and lower quantiles (*SI Appendix*, Fig. S8 *B*, *D*, *F*, *H*, *J*, and *L*). Using this approach, we determined that the greater morning peaks of *AhgCCA1* expression in autumn compared to those in spring was statistically significant under both light conditions tested (the shaded areas of the lower panels of Fig. 2 *E* and *F* represent the 95% CI of the difference between seasons not containing zero around dawn). During both sampling seasons, *AhgSIG5* transcripts reached peak abundance between the middle and end of the photoperiod (Fig. 2 *G* and *H*). This differs from *AtSIG5* transcript accumulation in *A. thaliana* under square-wave light/dark cycles under controlled conditions, where *AtSIG5* transcript abundance peaks around dawn (22). The peak of the diel fluctuation of *AhgSIG5* was significantly greater during the March sampling period than during the September sampling period (95% CI; Fig. 2 *G* and *H*). Transcripts encoding the SIG5 regulatory target *AhgpsbD* BLRP (Fig. 1*A*) had a diel fluctuation during the September sampling season that reached a much greater peak under shade conditions compared with sun conditions (Fig. 2 *I* and *J*), whereas there was very little diel fluctuation of *AhgpsbD* BLRP during the March sampling season.

When the diel dynamics of the pathway components were compared between sun and shade conditions (*SI Appendix*, Fig. S9), *AhgCCA1* transcript abundance was significantly greater under shade compared with sun conditions during the daytime, during both sampling seasons (95% CI; *SI Appendix*, Fig. S9 *E* and *F*). We did not identify the diminished *AtCCA1* oscillation that occurs under constant light with a very low R:FR (51) and on the shaded western side of crop fields around dawn (16). In comparison, *AhgSIG5* transcript abundance was not altered consistently between sun and shade conditions (95% CI; *SI Appendix*, Fig. S9 *G* and *H*). This tendency was also not observed for *AhgpsbD* BLRP transcript levels (95% CI; *SI Appendix*, Fig. S9 *I* and *J*).

### Dynamics of Environmental Regulation of the Signaling Pathway.

**Fig. 3.**
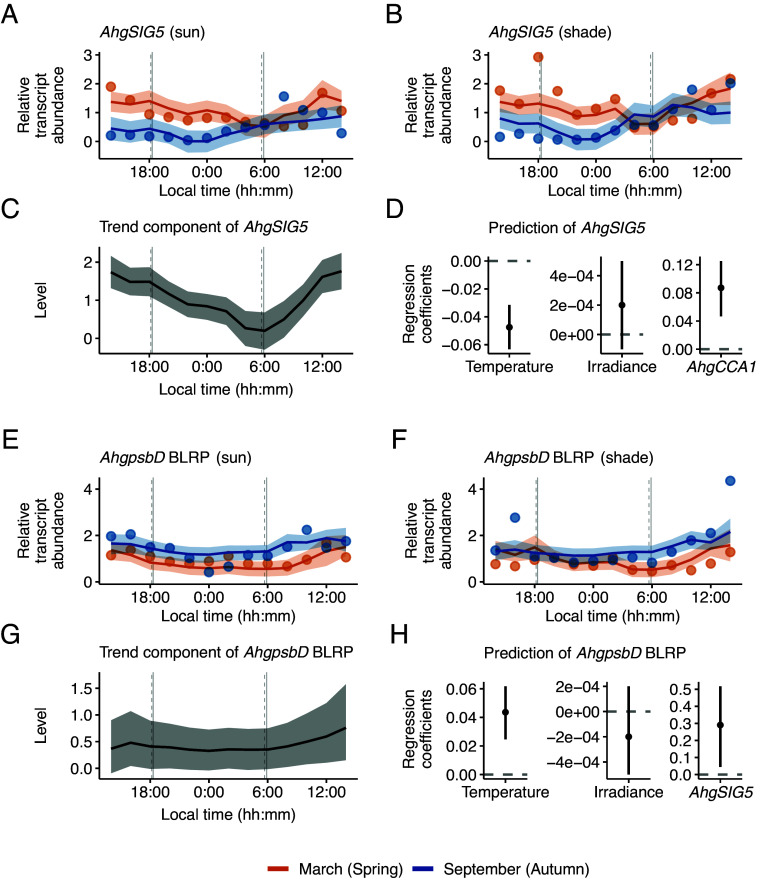
Ambient temperature and upstream regulatory components contribute to pathway dynamics in a natural population of *A. halleri*. (*A*–*H*) Bayesian estimation of the local level model with exogenous variables (LLMX) for transcript dynamics (in March and September 2015) of (*A*–*D*) *AhgSIG5* and (*E*–*H*) *AhgpsbD* BLRP. (*A*, *B*, *E*, and *F*) Modeled transcript dynamics (line and shaded area representing the median and the 95% CI of the posterior distribution, respectively; α, equations 10 to 13 in *SI Appendix*, *Supporting Text*) superimposed upon observed mean transcript abundance (circles). (*C* and *G*) Bayesian estimation of diel trend of each transcript (line and shaded area representing the median and the 95% CI, respectively; μ, equations 9). (*D* and *H*) Bayesian estimation of regression coefficient (β, equations 10 to 13) of environmental variables and potential upstream regulator (*AhgCCA1* for *AhgSIG5*, and *AhgSIG5* for *AhgpsbD* BLRP). Dots and error bars represent the median and the 95% CI, respectively. Vertical gray lines on time-series plots indicate the times of sunrise and sunset during March (solid line) and September (dashed). LLMX analysis used data from six replicate plants per condition.

We represented the behavior of the pathway components using a local level model with exogenous variables (referred to here as LLMX), a type of state-space modeling (52). This allows the inclusion of a circadian or diel trend within a regression model that predicts transcript levels from environmental variables and the upstream regulatory component (Fig. 1*A*). The output of the Bayesian estimation reproduced well the dynamics of the observed *AhgSIG5* transcript level (Pearson’s correlation coefficient = 0.77; Fig. 3 *A* and *B*), with convergence of MCMC sampling (*SI Appendix*, Fig. S10). The model estimated a clear diel trend of *AhgSIG5* transcript level that reached the trough level at dawn and the peak level between the middle and end of the photoperiod (Fig. 3*C*). The model also estimated significant negative and positive effects of ambient temperature and *AhgCCA1* on *AhgSIG5* transcript abundance, respectively (the error bars representing the 95% CI of regression coefficients not containing zero; Fig. 3*D*). Irradiance may positively affect the estimation of *AhgSIG5* transcript abundance; however, this effect was not significant in this analysis (Fig. 3*D*). Under controlled laboratory conditions, a temperature reduction leads to an increase in *SIG5* transcript abundance (33). Therefore, the estimated negative relationship between ambient temperature and *AhgSIG5* transcript levels (Fig. 3*D*) could suggest similar regulation in *A. halleri* under naturally fluctuating conditions. If the temperature input to the model was set to a constant value (the mean temperature across all conditions) and the estimated parameter values in this model were used, the greater *AhgSIG5* transcript abundance during March disappeared, and the estimated phase was slightly altered (compare *SI Appendix*, Fig. S11 *A*–*D*). This suggests that temperature is a key predictor of the difference in *AhgSIG5* transcript abundance between seasons. Setting the irradiance input to a constant level (the mean irradiance across all conditions) did not affect *AhgSIG5* dynamics (compare *SI Appendix*, Fig. S11 *A*, *B*, *E*, and *F*). Setting a constant *AhgCCA1* transcript level shifted the estimated phase of *AhgSIG5* dynamics with the exception of sun conditions during March (compare *SI Appendix*, Fig. S11 *A*, *B*, *G*, and *H*), suggesting that *AhgCCA1* input contributes to the prediction of the *AhgSIG5* oscillation.

Diel fluctuations of *AhgpsbD* BLRP transcript abundance were reproduced reasonably well by the model (Pearson’s correlation coefficient = 0.68; Fig. 3 *E* and *F*), with convergence of MCMC sampling (*SI Appendix*, Fig. S12). The model predicted a diel trend of *AhgpsbD* BLRP transcript level that increased toward the end of the photoperiod (Fig. 3*G*). The model estimated a significant positive effect of ambient temperature on *AhgpsbD* BLRP transcript abundance (95% CI; Fig. 3*H*). In *A. thaliana* under controlled conditions, *psbD* BLRP transcript accumulation is regulated by SIG5 (21), and within our model, there was also a significant positive coefficient of regression between *AhgSIG5* and *AhgpsbD* BLRP transcript levels (95% CI; Fig. 3*H*). When the temperature input was set to a constant value (the mean temperature among all conditions) with estimated parameter values in this model, greater *AhgpsbD* BLRP transcript abundance in September disappeared, the diel dynamics became slightly flatter, and the estimated phase under sun conditions was altered (compare *SI Appendix*, Fig. S13 *A*–*D*). This suggests that temperature is a key predictor of the difference in *AhgpsbD* BLRP transcript abundance between seasons. Constant irradiance did not affect the *AhgpsbD* BLRP dynamics (compare *SI Appendix*, Fig. S13 *A*, *B*, *E*, and *F*). Constant *AhgSIG5* transcript level caused flatter *AhgpsbD* BLRP dynamics (compare *SI Appendix*, Fig. S13 *A*, *B*, *G*, and *H*) and changed the estimated phase of the model output, suggesting that *AhgSIG5* input contributes to the prediction of the *AhgpsbD* BLRP oscillation.

Overall, this analysis identifies that the ambient temperature, rather than the irradiance, was important for predicting the seasonal differences in the transcript abundance of *AhgSIG5* and *AhgpsbD* BLRP under naturally fluctuating conditions. Irradiance may affect the transcript abundance under some circumstances, such as in dark adapted seedlings (23). In addition, the circadian clock (*AhgCCA1*) affected the diel oscillation of *AhgSIG5* transcript abundance (Fig. 3*D* and *SI Appendix*, Fig. S11 *G* and *H*), and *AhgSIG5* affected the diel oscillation of *AhgpsbD* BLRP transcript abundance (Fig. 3*H* and *SI Appendix*, Fig. S13 *G* and *H*). More specifically, the seasonal difference in temperature affected the base levels of *AhgSIG5* and *AhgpsbD* BLRP transcripts, while the diel oscillation patterns of *AhgSIG5* and *AhgpsbD* BLRP transcripts were affected by the levels of their upstream regulators rather than diel temperature fluctuations.

The significant effects of temperature on *AhgSIG5* and *AhgpsbD* BLRP transcripts (Fig. 3 *D* and *H*), and the restriction to specific periods of the day of significant differences between the transcript dynamics under the different temperature conditions of March and September (Fig. 2 *E*–*J*), is reminiscent of the concept of circadian gating. Circadian gating is the process whereby the circadian clock restricts certain biological processes to specific times in the 24 h cycle (53). In plants, this can take the form of a circadian rhythm in the magnitude of the response to identical environmental stimuli given at different times of day (8).

### Temporal Gating of Temperature Regulation of SIG5-Mediated Signaling to Chloroplasts under Natural Conditions.

**Fig. 4.**
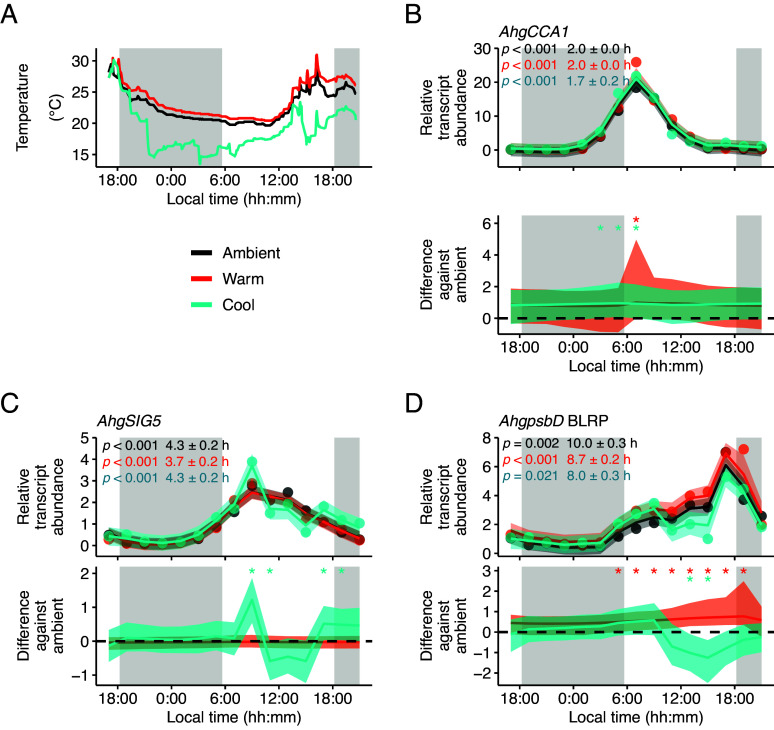
Estimation of diel rhythms of temporal gating of temperature responses in a natural population of *A. halleri*. (*A*) Temperature changes in each condition of temperature manipulation experiments in September 2016. (*B*–*D*) Bayesian estimation of STM for transcript dynamics of (*B*) *AhgCCA1*, (*C*) *AhgSIG5,* and (*D*) *AhgpsbD BLRP*. In each panel, the upper graphs show the predicted relative transcript abundance for ambient (black; $\mu_{1}$, equation 1 in *SI Appendix*, *Supporting Text*), warm (red; $\mu_{2}$, equation 3) and cool (light blue; $\mu_{3}$, equation 7) conditions with the mean of observed values (dots), and the lower graphs represent the differences in transcript abundance against the ambient condition (δ, equation 2; $\delta_{2}$, equation 6). In each graph, the solid line and the shaded region are the median and the 95% CI of the posterior distribution. When the 95% credible interval of the difference between conditions does not contain zero, the difference is considered significant. Panels *B*–*D* include estimation of rhythmicity and peak time relative to solar dawn of underlying data, using JTK_CYCLE. Shaded boxes on *A*–*D* indicate the period between sunset and sunrise. STM analysis used data from six replicate plants.

Our LLMX analysis suggests that under natural conditions, lower ambient temperatures might up-regulate *AhgSIG5* transcript levels, and greater ambient temperatures might up-regulate *AhgpsbD* BLRP transcript levels (Fig. 3 *D* and *H*). To test this hypothesis and further examine whether a circadian gating-like process might occur, we applied moderate temperature manipulations to adjacent patches of *A. halleri* plants, in the field, using custom-designed equipment (Fig. 4*A* and *SI Appendix*, Fig. S14 *A*–*D*). We collected 24-h time-series of RNA samples from these plant patches (*SI Appendix*, Fig. S14 *E*–*G*) and interpreted the data with STMs (Fig. 4 *B*–*D*). This experiment occurred during September 2016, to take advantage of the greater magnitude of oscillation of the transcripts observed during experimentation during September 2015 compared with March 2015 (Fig. 2 *E*–*J*). *AhgCCA1* transcripts underwent a substantial fold-change fluctuation over the 24 h cycle, ranging from 210-fold (moderate temperature reduction) to 339-fold (ambient temperature) and 1,230-fold (moderate temperature increase) (*SI Appendix*, Fig. S14*E*). Interestingly, a moderate temperature reduction treatment during September 2016 did not decrease the fluctuation of *AhgCCA1* transcripts to a level equivalent to the March 2015 sampling season (fivefold to sevenfold fluctuation), suggesting that either there is a tipping point temperature below which *A. halleri CCA1* transcripts have a substantial loss of rhythmicity, or that the past temperature history of the plants during each season can influence the amplitude of oscillation of this clock transcript.

The STM reproduced well the dynamics of the observed transcript levels (Pearson’s correlation coefficient = 0.99 for *AhgCCA1*, 0.98 for *AhgSIG5*, and 0.98 for *AhgpsbD* BLRP). To further evaluate the model fit, we performed a residual analysis (*SI Appendix*, Fig. S15). Residuals against the fitted values showed no specific trend around zero, supporting the validity of the model (*SI Appendix*, Fig. S15 *A*, *C*, and *E*). In support of the model, quantile–quantile plots of the residuals showed that they generally follow normal distributions, except for deviations from the theoretical lines at higher and lower quantiles (*SI Appendix*, Fig. S15 *B*, *D*, and *F*). Both the moderate temperature increase and temperature reduction treatment caused a small significant upregulation of *AhgCCA1* transcript abundance around dawn, relative to the ambient temperature control (significance at >95% probability; Fig. 4*B*). The moderate temperature increase treatment was without effect upon *AhgSIG5* transcript abundance (Fig. 4*C*). In comparison, the temperature reduction treatment up-regulated *AhgSIG5* transcripts significantly after dawn and around dusk, relative to the ambient temperature control (significance at >95% probability; Fig. 4*C*). This is consistent with the negative coefficient of regression of temperature on *AhgSIG5* transcript under naturally fluctuating conditions (Fig. 3*D*), and with the upregulation of *A. thaliana SIG5* by a short cold treatment under laboratory conditions (21, 33). The restriction of the response of *AhgSIG5* transcripts to the moderate temperature reduction to certain times of day (Fig. 4*C*) again supports the notion that there is temporal gating of the response of *AhgSIG5* to temperature under naturally fluctuating conditions. The peak time of *AhgSIG5* occurred later in September 2016 (4.3 h after dawn under the ambient condition) compared with September 2015 (2 h after dawn under the sun condition). This might be partly due to the later temperature trough and peak times in 2016; it was coldest at 11:00 in 2016 and at 04:00 in 2015, and warmest at 16:00 in 2016 and at 14:00 in 2015 (compare Figs. 2*A* and 4*A*).

Transcripts for the chloroplast target of SIG5, *AhgpsbD* BLRP, were also altered by temperature manipulation (Fig. 4*D*). The moderate temperature elevation significantly increased *AhgpsbD* BLRP transcript levels relative to the control, whereas the moderate temperature reduction significantly reduced *AhgpsbD* BLRP transcripts relative to the control (significance at >95% probability; Fig. 4*D*). This is consistent with the positive coefficient of regression of temperature on *AhgpsbD* BLRP transcript abundance under naturally fluctuating conditions (Fig. 3*H*). The response of *AhgpsbD* BLRP transcripts to temperature manipulations was restricted to the photoperiod, potentially because chloroplast DNA binding and transcription by PEP generally requires light (54–58). The peak time of *AhgpsbD* BLRP transcripts occurred later in September 2016 (10 h after dawn under the ambient condition) compared with September 2015 (8.2 h after dawn under the sun condition). This might be partly due to the later temperature trough and peak times in 2016, similar to *AhgSIG5* described above.

To assess the validity of our LLMX model established using the 2015 data, we predicted the transcript abundance of *AhgSIG5* and *AhgpsbD* BLRP in the 2016 experiment, using the LLMX model with estimated parameters, and the 2016 data as explanatory variables (temperature, irradiance, and transcript abundance of upstream genes). The model predicted that *AhgSIG5* transcripts would peak in the morning, which was similar to the observed data (*SI Appendix*, Fig. S16 *A*, *Upper*). *AhgSIG5* transcript abundance was predicted to have a higher level under the cooled condition around dawn and dusk, which is consistent with the observed data (*SI Appendix*, Fig. S16 *A*, *Lower*). Under the warmed condition, *AhgSIG5* was predicted to have a higher abundance after dawn and be lower at most other time points (*SI Appendix*, Fig. S16 *A*, *Lower*). *AhgpsbD* BLRP transcript peaked in the early afternoon under the ambient condition, which is slightly earlier than that in the observed data (*SI Appendix*, Fig. S16 *B*, *Upper*; Fig. 4*D*). Under the cooled condition, *AhgpsbD* BLRP transcript abundance was predicted to be lower during the afternoon, which is similar to the observed data (*SI Appendix*, Fig. S16 *B*, *Lower*), and was predicted to be greater under the warm condition after dawn and around dusk (*SI Appendix*, Fig. S16 *B*, *Lower*). The discrepancy in the peak timing between the model predictions (derived from the 2015 data) and the 2016 data might be attributed to the influence of environmental factors that are not included in the current model, such as humidity and precipitation. The incorporation of time-varying regression coefficients into the model may facilitate the reproduction of temporal gating of the response of genes to temperature during the photoperiod.

### Evaluation of Causal Regulation within a Circadian-Regulated Signaling Pathway under Natural Conditions.

We performed CCM analysis of the relationship between *AhgCCA1* and *AhgSIG5*, and of the relationship between *AhgSIG5* and *AhgpsbD* BLRP, using a variety of time-delays between each pair of variables. We considered potential time-delays in CCM analysis (59) because the abundance of each related transcript was monitored at each timepoint, but their responses to each other might not be instantaneous. For example, in *A. thaliana* under controlled square-wave light/dark cycle conditions, *AtCCA1* transcript abundance peaks at dawn, *AtSIG5* approximately 3 h after dawn, and *AtpsbD* BLRP approximately 6 h after dawn (22).

**Fig. 5.**
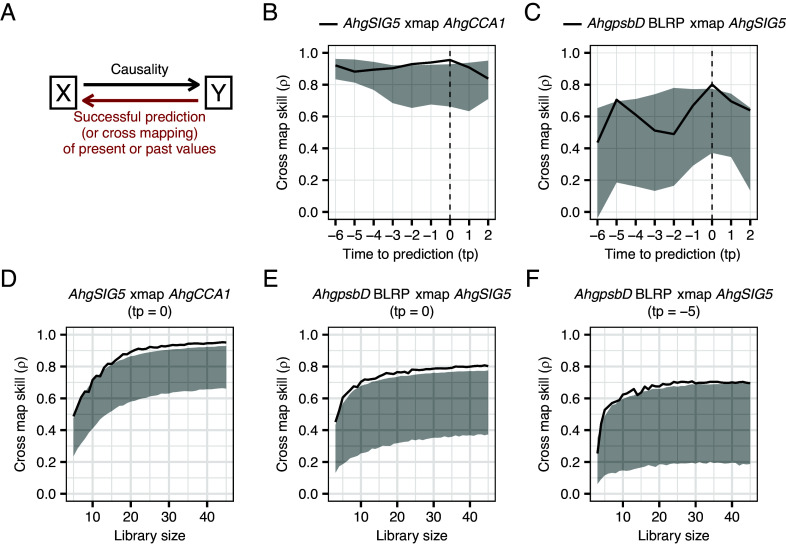
Evaluation of causal relationships between components of a circadian signaling pathway in a natural population of *A. halleri*. (*A*) The direction of causality (from *X* to *Y*) and that of prediction (cross mapping) in CCM (from *Y* to *X*) is opposite. (*B* and *C*) Estimation of causality between pairs of pathway components, across a range of time delays between the pathway component (time to prediction, tp) for temperature manipulation experiments in September 2016. Cross-map skill (ρ) provides a measure of the potential causality strength between the two variables. (*D*–*F*) Test of convergence, i.e., an improvement in cross-map skill according to increase in a library size (number of time points used to reconstruct a state space), for each time lag with a significant cross-map skill. In *B*–*F*, the solid line represents the cross-map skill between pathway components, and shaded area represents the 95% interval of the cross-map skill using 1,000 diel surrogate time series as the explanatory variable that reflects the same degree of oscillation but with the sequence of variation randomized (i.e., null model).

To conduct CCM analysis upon data from the temperature manipulation experiments in September 2016, we first determined the optimal embedding dimension for each variable (*AhgCCA1*, *AhgSIG5,* and *AhgpsbD* BLRP; *SI Appendix*, Fig. S17). A feature of CCM analysis that can confuse readers is that the direction of successful prediction is *opposite* to that of causality (Fig. 5*A*). CCM analysis identified a significant prediction (cross-map skill) of *AhgCCA1* transcript abundance from *AhgSIG5* transcript abundance (Fig. 5*B*) when no time delay was incorporated between the variables. Furthermore, a significant prediction of *AhgSIG5* from *AhgpsbD* BLRP occurred when no time delay was incorporated, and potentially also with a time delay between *AhgSIG5* and *AhgpsbD* BLRP (Fig. 5*C*). In both cases, the cross-map skill exceeded a 95% interval of the diel surrogate, which imitates the same degree of oscillation of the explanatory variable, but where the variation is randomized (Fig. 5 *B* and *C*). When the library size (number of time points used to reconstruct a state space) was increased, cross-map skill was improved for each time lag, attaining a significant cross-map skill (Fig. 5 *D*–*F*), suggesting convergence (a key property that distinguishes causation from simple correlation) (45).

We extended our analysis to the September 2015 data, to evaluate the robustness of these findings. Significant predictions (supporting causality) were detected with slight differences in time delays (*SI Appendix*, Fig. S18). When the analysis was also performed on the March 2015 data, the predictions were worse than for September 2015 and September 2016 (*SI Appendix*, Fig. S19). Nevertheless, a significant prediction of *AhgCCA1* from *AhgSIG5* was detected with a time delay (*SI Appendix*, Fig. S19). The failure to detect causality from *AhgSIG5* to *AhgpsbD* BLRP in the March 2015 data might be due to the weak oscillation of the transcripts, particularly *AhgpsbD* BLRP (Fig. 2 and *SI Appendix*, Fig. S9).

Overall, CCM analysis suggests that there is a causal relationship between fluctuations in *AhgCCA1* and *AhgSIG5*, and *AhgSIG5* and *AhgpsbD* BLRP, and that there is a potential time-delay from nuclear genome-encoding *AhgSIG5* to chloroplast genome-encoding *AhgpsbD* BLRP in a natural plant population.

### Universality of Circadian and Environmental Signal Integration in Natural Environments.

To investigate whether our LLMX models of circadian and environmental signal integration operate consistently across seasons with different photoperiods, we applied our modeling framework to existing RNA-seq data from four 48-h time-series collected during 2013 (12). *AhgSIG5* transcript dynamics were predicted successfully for samples collected at the spring equinox, summer solstice, autumn equinox, and winter solstice (*SI Appendix*, Fig. S20). The model estimated negative, positive, and positive effects of ambient temperature, irradiance, and *AhgCCA1* on *AhgSIG5* transcript abundance, respectively (*SI Appendix*, Fig. S20*F*). The effects of temperature and irradiance were not significant and significant, respectively (*SI Appendix*, Fig. S20*F*), in contrast to the 2015 data (Fig. 3*D*), suggesting that their significance varies with environmental variations or that previous normalization of these RNA-seq data attenuated some sensitivity. Overall, this indicates that the modeling framework can predict pathway outputs under seasonally different photoperiods.

**Fig. 6.**
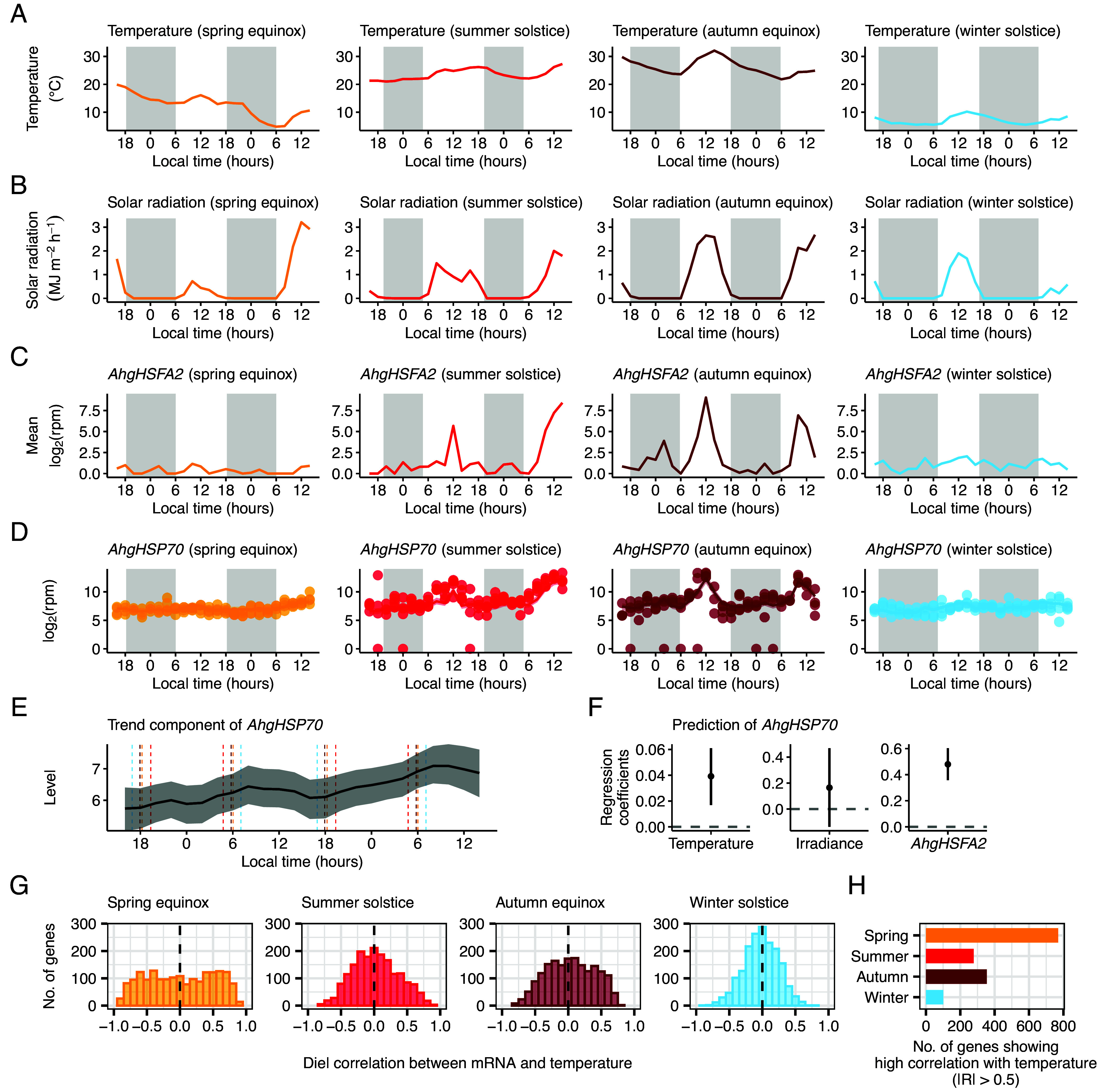
Hundreds of genes undergo circadian and environmental signal integration in a natural population of *A. halleri*. (*A*–*F*) Prediction of *AhgHSP70* signaling pathway dynamics in four seasons with different day length. (*A* and *B*) Diel fluctuations in (*A*) ambient temperature and (*B*) solar radiation, at 2-h intervals in the four sets of 48-h sampling period in 2013. (*C*) *AhgHSFA2* transcript abundance averaged over four biological replicates during the sampling period. (*D*) Bayesian estimation of the local level model with exogenous variables (LLMX) for *AhgHSP70* transcript dynamics during the sampling period. Modeled transcript dynamics (lines and shaded area representing the median and the 95% CI of the posterior distribution, respectively) are superimposed upon observed mean transcript abundance (circles). (*E*) Bayesian estimation of trend component of *AhgHSP70* transcript (line and shaded area representing the median and the 95% CI, respectively). Vertical dashed lines indicate the times of sunrise and sunset in each season. (*F*) Bayesian estimation of regression coefficient of environmental variables and a potential upstream regulator (*AhgHSFA2*). Dots and error bars represent the median and the 95% CI, respectively. (*G*) A frequency distribution of diel correlation between mRNA levels of oscillating genes and ambient temperature in each season. (*H*) Number of genes showing high correlation with ambient temperature (|R| > 0.5) in each season. Shaded boxes on (*A*–*D*) indicate the period between sunset and sunrise. LLMX analysis used data from four replicate plants per season.

To understand the universality of our model, we applied the same approach to other signaling pathways that are thought to be influenced by environmental factors. One of the best examples is the upregulation of heat shock proteins by heat shock factors, triggered upon exposure to heat stress (60–62). Using existing RNA-seq data (12), our model could successfully predict *HEAT SHOCK PROTEIN70* (*AhgHSP70*) transcript dynamics from environmental variables and an upstream regulatory component *HEAT SHOCK TRANSCRIPTION FACTOR A2* (*AhgHSFA2*) (Fig. 6 *A*–*F*) (12). The model estimated positive effects of ambient temperature and *AhgHSFA2* on *AhgHSP70* transcript abundance (Fig. 6*F*), which aligns with previous studies (63, 64).

As a slightly more complex pathway, we extended our approach to the C-REPEAT/DRE BINDING FACTOR (CBF) pathway, which is critical for cold acclimation and gated by the circadian clock (60, 65). Clock regulation of the *CBF*s in *A. thaliana* involves multiple clock components including CCA1, PRR7, and the RVE family (66–68). Our model could successfully predict *COLD-REGULATED15A* (*AhgCOR15A*) transcript dynamics from environmental variables, *AhgCCA1* and the upstream regulatory component *AhgCBF1*, from RNA-seq data (12) (*SI Appendix*, Fig. S21). The model estimated negative, positive, and positive effects of ambient temperature, irradiance, and *AhgCBF1* on *AhgCOR15A* transcript abundance, respectively (*SI Appendix*, Fig. S21*G*). The effect of *AhgCCA1* was not significant, suggesting that control of *AhgCOR15A* by *AhgCCA1* is relatively minor compared to its regulation by *AhgCBF1* in plants under natural conditions (*SI Appendix*, Fig. S21*G*), with other clock components having potentially competing roles (66–68). Aside from this, these model predictions are consistent with previous studies (65, 66). This suggests our modeling framework can be applied to other questions concerning the integration of circadian and environmental signals.

These results suggest that the integration of circadian and environmental signals is a common feature of several signaling pathways. To gain insight into how many oscillating genes are also regulated by temperature, a prominent environmental signal, we calculated the diel correlation between the abundance of these oscillating transcripts and ambient temperature (Fig. 6*G*). The results showed that the number of genes with a high positive or negative correlation with temperature was greatest in spring, with 770 out of 1,804 diel oscillating genes (q value < 0.05, JTK_CYCLE) having an absolute correlation coefficient greater than 0.5 (Fig. 6*H*). Conversely, the number was smallest in winter, with only 101 genes having an absolute correlation coefficient greater than 0.5 (Fig. 6*H*). Thus, hundreds of genes with a diel oscillation are likely affected by temperature, although this regulation is diminished in winter.

## Discussion

We deployed a set of approaches that provided insights into the integration and transduction of circadian and environmental signals in a natural plant population. Using a modeling approach, we predicted that the circadian clock and ambient temperature are potential regulators of *AhgSIG5*-mediated signaling to chloroplasts, in a natural population of *A. halleri*. There was a significant influence of *AhgCCA1* on *AhgSIG5* transcript abundance, and of *AhgSIG5* on *AhgpsbD* BLRP transcript abundance (Fig. 3 *D* and *H*). Furthermore, CCM analysis predicted significant causal relationships within this signaling pathway in the field (Fig. 5 and *SI Appendix*, Figs. S18 and S19). Therefore, it appears that in a natural plant population, a signal is communicated from the circadian oscillator (using *AhgCCA1* as a proxy) to the signaling pathway output of *AhgpsbD* BLRP. One interpretation is that the pathway couples the circadian oscillator and temperature response processes to chloroplast gene transcription under naturally fluctuating conditions. The lack of causality between *AhgSIG5* and *AhgpsbD* BLRP during the March 2015 season could suggest environmentally induced variation in coupling strength between these pathway components. This is consistent with the notion that SIG5-independent mechanisms might regulate *psbD* BLRP (33), and/or of a threshold level of SIG5 activity that is necessary for its transcriptional control of *psbD* BLRP (22).

Under field conditions, *AhgSIG5* oscillated with a later phase compared with its phase in *A. thaliana* under controlled conditions using a square-wave light–dark cycle (22) (e.g., Fig. 2 *G* and *H* and *SI Appendix*, Fig. S20*D*). This suggests that under controlled conditions, the transcript undergoes an acute response to the instantaneous dawn that is absent under naturally fluctuating conditions. This difference in *SIG5* dynamics between controlled and field conditions is reminiscent of differences in diel dynamics of some plant circadian clock transcripts under controlled versus natural conditions (12, 14). Similarly, there are substantial differences between the phases of clock-controlled processes under controlled and natural conditions for other organisms, including *Drosophila*, subterranean rodents, and humans (69–71).

We identified seasonal differences in the maximum accumulation of *AhgCCA1*, *AhgSIG5,* and *AhgpsbD* BLRP. The difference in *AhgCCA1* dynamics between these sampling seasons likely reflects the decreased amplitude of the circadian oscillator that occurs under lower temperature conditions, in both controlled laboratory environments and the field (12, 72–74). The difference in the seasonal response of *AhgSIG5* compared with *AhgCCA1* suggests that an additional temperature input into this pathway occurs between the circadian oscillator and *AhgSIG5*. In *A. thaliana*, *AtSIG5* transcripts are up-regulated by short cold temperature treatments (21, 33). In our field experiment, *AhgSIG5* transcript accumulation showed a negative coefficient of regression with temperature (Fig. 3*D*), with its accumulation increased during the morning and around dusk by a low temperature manipulation (Fig. 4*C*). This suggests that under lower temperature conditions, *AhgSIG5* transcript abundance will increase. Therefore, the lower temperatures of the spring sampling season compared with the autumn sampling season (Fig. 2 *A* and *B*) might explain the significantly greater abundance of *AhgSIG5* transcripts at many times of day during the spring. A further feature of the data is the relative robustness of the dawn timing of peak *AhgCCA1* abundance, irrespective of the environmental conditions (Fig. 2 *E* and *F*), which is consistent with a previous field study (12). In *Arabidopsis*, *AtCCA1* is light-induced (75, 76), yet under field conditions *AhgCCA1* reached a peak around astronomical dawn when the irradiance was relatively low, and was unresponsive to increased light intensity later in the day (Fig. 2 *C*–*F*). Furthermore, its peak time was relatively unaffected by seasonal differences in light conditions (Fig. 2 *C*–*F*). This reveals an astonishing stability of the phase of expression of a clock gene in plants under complex fluctuating environments, which might be accomplished through the complexity of the *Arabidopsis* circadian oscillator that is thought to confer robustness to its regulation (77, 78), and perhaps a restriction of light-induced phase shifts to certain times of day due to the gating of light inputs. Mathematical modeling indicates that the interplay between different simultaneous circadian entrainment cues might reinforce circadian clocks (79), although the timing of individual cues applied in combination can also cause changes in the phase (80). The processes that align the phase of the plant circadian clock with the growing environment, under naturally fluctuating conditions, represents a fascinating topic for future investigation.

Because we considered *AhgpsbD* BLRP to represent the ultimate output from the signaling pathway (Fig. 1*A*), our analysis suggests that environmental inputs occurred within at least three positions in the pathway; first, in the regulation of *AhgCCA1* transcript accumulation by the season or temperature, second, in the regulation of *AhgSIG5* transcript accumulation by temperature, and third, in the regulation of *AhgpsbD* BLRP transcript accumulation by temperature. These environmental inputs might occur through biologically independent processes. One possibility is temperature inputs to the circadian clock mediated by temperature-responsive components, such as the evening complex (81, 82). A further mechanism could be the regulation of *AhgSIG5* by HY5, which is a known regulator of SIG5 that participates in low-temperature gene regulation (21, 27, 83) and underlies a response of *AtSIG5* transcripts to cold in *A. thaliana* (33). Furthermore, there might be direct effects of light upon sigma factor activity in chloroplasts through, for example, redox regulation (84) or light- and temperature-regulation of chloroplast protein import (85). We did not consider here the short- or long-term history of light or temperature conditions upon leaves prior to experimentation. However, the past temperature conditions can affect *AhgCCA1* oscillations, since the sensitivity of *AhgCCA1* oscillations to a moderate temperature reduction treatment during September 2016 was limited compared with its response to the lower ambient temperature conditions during Spring 2015.

Statistical modeling of the transcriptome of field-grown *Oryza sativa* (rice) concluded that the main environmental driver of *OsSIG5* (*Os05g0586600*) transcript accumulation is temperature (9). In this case, the temperature had a negative regression coefficient with *OsSIG5* (9). This is consistent with our finding of a negative coefficient of regression of temperature on *AhgSIG5*. The study of the rice transcriptome in the field (9) did not monitor chloroplast-encoded transcripts, so a direct comparison between rice *psbD* BLRP and our data is not possible.

We detected a 24 h fluctuation of the influence of the temperature upon each of the three genes (Fig. 4). This finding from the field is corroborated by laboratory experiments, which demonstrate that the circadian oscillator gates its own response to temperature (86), and the response of *AtSIG5* to blue light and cold temperatures is gated by the circadian oscillator (22, 33). Statistical modeling of the rice transcriptome in the field identified 24 h oscillations in the coefficient that describes the relationship between temperature and transcript levels, which is also reminiscent of circadian gating (9). In field experiments, it is difficult to link fluctuating temperature sensitivity to the circadian oscillator causatively, although a combination of field and laboratory experiments investigating the environmental regulation of *FLOWERING LOCUS T* (*FT*) expression suggests that the circadian clock can gate light quality and temperature responses of *FT* under field conditions (18, 87). Taken together, this suggests that processes leading to circadian gating might operate in natural plant populations to modulate their environmental responses.

The molecular aspects of circadian signaling under field conditions represent a relatively understudied topic. Here, we adopted quantitative approaches to interpret relatively noisy transcript data collected under complex fluctuating environments, to investigate the functioning of a specific pathway. This allowed us to infer multiple positions of environmental inputs into the pathway, and temporal gating of a response to temperature. Our approaches provide a framework to study environmental signal integration in all organisms under field conditions, can accommodate moderate changes in photoperiod length, and can be applied to a variety of signaling pathways. The models could be adapted to incorporate additional environmental variables, with care being necessary to ensure that the variables do not overlap (e.g., total irradiance and blue light overlap). In future, it would be informative to examine whether physiological or behavioral outputs can be predicted by our modeling framework, although approaches such as machine learning might provide an alternative for predicting the dynamics of pathways that are more complex than those examined here. Our approaches could be important to understand circadian-regulated processes in an increasingly unpredictable climate.

## Materials and Methods

### Field Site, Tissue Sampling, and Environmental Monitoring and Manipulation.

We used a naturally occurring population of *A. halleri* subsp. *gemmifera* (Matsum.) O'Kane & Al-Shehbaz growing beside a forested stream in Hyogo Prefecture, Japan (12, 43, 88) (Fig. 1*B*). Time-series of tissue for RNA isolation were obtained from these plants during several sampling seasons, with simultaneous monitoring of irradiance and temperature. During one sampling season, the temperature was manipulated around patches of plants. For all details, see *SI Appendix*, *Supporting Text*.

### RNA Isolation and RT-qPCR.

Leaf tissue preserved in RNA*later* Stabilization Solution (Thermo Fisher Scientific, Waltham, MA) was used for subsequent kit-based RNA isolation and RT-qPCR. Full details including gene homology and primer design are in *SI Appendix*, *Supporting Text*.

### STM.

The STM allows inference of a trend within time-series data that contains both sampling noise and biological stochasticity, and allows a level of statistical confidence to be applied to that trend. Model details, underlying equations, and evidence for model convergence are in *SI Appendix*, *Supporting Text*.

### Local Level Model with Exogenous Variables.

A local level model with exogenous variables (LLMX) was used to analyze a diel trend and the effect of environmental variables on transcript abundance. The LLMX models were applied to both our RT-qPCR data (Fig. 3), and existing RNA-seq data (12, 44). Model details, underlying equations, and evidence for model convergence are in *SI Appendix*, *Supporting Text*.

### CCM.

CCM (45) was used to evaluate causality between signaling pathway components. Details of determination of the embedding dimension and CCM execution is in *SI Appendix*, *Supporting Text*.

## Supplementary Material

Appendix 01 (PDF)

Dataset S01 (XLSX)

## Data Availability

All study data are included in the article and/or supporting information. Code for modeling is provided at https://github.com/hnishio/SIG5_field_PNAS.git (89).
