# Supplementary material for: Circadian and environmental signal integration in a natural population of Arabidopsis
Source: Proc Natl Acad Sci U S A. 2024 Aug 22;121(35):e2402697121. doi: 10.1073/pnas.2402697121 (PMC11363283; doi:10.1073/pnas.2402697121)
Supplement: Supplementary file 1 — Appendix 01 (PDF) [file pnas.2402697121.sapp.pdf]

PNAS

# Supporting Information for
Circadian and environmental signal integration in a natural population of *Arabidopsis*

Haruki Nishio*, Dora L. Cano-Ramirez*, Tomoaki Muranaka, Luíza Lane de Barros Dantas, Mie N. Honjo, Jiro Sugisaka, Hiroshi Kudoh, Antony N. Dodd.

* Equal contribution

Email: antony.dodd@jic.ac.uk

**This PDF file includes:**

Supporting Text
Figures S1 to S22
Tables S1 to S3
SI References

**Other supporting materials for this manuscript include the following:**

Datasets S1

**Supporting Information Text - Extended Materials and Methods**

*Field site and plant material*

Our experiments used a naturally occurring population of *Arabidopsis halleri* subsp. *gemmifera* (Matsum.) O'Kane & Al-Shehbaz growing beside a forested stream in Hyogo Prefecture, Japan (Omoide-gawa site; 35°06’ N, 134°55’ E, elevation 190–230 m) (1-3) (Fig. 1B). We selected *A. halleri* as an experimental model for several reasons (4). First, it has high nucleotide sequence identity and good synteny with *A. thaliana* (5). Second, unlike *A. thaliana*, the perennial life history of *A. halleri* allows investigation of transcriptional responses across the seasons (3, 6). Many individuals are clones because the species propagates by producing clonal rosettes as well as by seeds, which allows repeated sampling from single genotypes. Furthermore, *A. halleri* is metal tolerant and occurs in natural habitats that are relatively free from other vegetation due to contamination by heavy metals, which provides experimentally-convenient sites enriched with many *A. halleri* plants (3). *Arabidopsis halleri* subsp. *gemmifera* at this site was previously identified by examination of museum and herbarium specimens, and a nearby population provided material for sequencing the *A. halleri* genome (3, 5).

*AhgCCA1* (*g097040*) and *AhgSIG5* (*g25274*) were identified from *A. halleri* genome Version Ahal2.2 (5). *AhgCCA1* has 94.8% coding sequence identity and 93.3% protein sequence identity with *AtCCA1*. *AhgSIG5* has 94.9% coding sequence identity and 95.0% protein sequence identity with *AtSIG5*. Chloroplast-encoded *psbD* is not annotated within Version Ahal2.2 of the *A. halleri* genome, and we identified this instead within scaffold 2 of an *A. halleri* reference transcriptome (7). The *AhgpsbD* BLRP promoter region, which was our focus, has a 100% sequence identity with *AtpsbD* BLRP (8).

*Experimental conditions for field sampling of A. halleri*

The first sets of samples were obtained under natural conditions without temperature manipulation during 24 – 26 March 2015 and 15 – 17 September 2015, which were close to the spring and autumn equinox at the field site, respectively. We exploited variations in environmental

conditions across the field site, and sampled leaves from the locations nominated as "sun" and "shade" sites on successive days. At "sun" locations, plants received direct sunlight during the day, and at "shade" locations plants received sunlight filtered by surrounding vegetation for most of the day with the sites identified by measurement of the ratio of red to far red light (*SI Appendix*, Fig. S4; R:FR calculated as the photon irradiance from 660 to 670nm divided by the photon irradiance from 725 to 735nm (9)). During March 2015, plants received more direct sunlight, whereas during September 2015 the light was scattered through sky overcast with clouds in combination with persistent rain.

The second sets of samples were obtained under natural conditions with manipulation of the temperature conditions around patches of plants during 13 – 14 September 2016, which was close to the autumn equinox at the field site. In addition to control plants that were not manipulated (*SI Appendix*, Fig. S14A), we applied two temperature treatments. These were (1) a continuous temperature increase (*SI Appendix*, Fig. S14B), whereby plants were covered with clear plastic horticultural domes to block air currents and trap warm air; (2) a continuous temperature reduction, using a custom device that passed air through a duct within a heat-exchanging ice-filled polystyrene box and expelled the chilled air into a clear horticultural dome covering the plants, with chilling augmented by small ice packs within the dome (*SI Appendix*, Fig. S14C).

*Field sampling for transcript analysis*

Across all experimental conditions, the same sampling and RNA isolation procedures were used. At 2-hour intervals, a fully expanded rosette leaf was excised with dissecting scissors from 6 replicate plants for each condition. The time-courses using naturally occurring sun and shade conditions each comprised 13 sampling timepoints over a total of 26 hours (from 14:00 on the first day to 16:00 on the second day). The time-courses involving artificial temperature manipulations comprised 15 sampling timepoints over a total of 30 hours (from 17:00 on the first day to 21:00 on the second day). The same replicate plants were sampled repeatedly through each time-series, but different plant patches were used in different sampling seasons. Sampled leaves were placed

immediately into individual microtubes containing at least 400 μL RNA*later* Stabilization Solution (Thermo Fisher Scientific, Waltham, MA, USA). Scissors and forceps were cleaned with 70% (w/v) ethanol between samples. After sampling, tubes were placed temporarily on dry ice for up to 2 hours, at -40 °C for 3 days in a portable freezer during transfer to the laboratory, and then at -80 °C until RNA isolation. During hours of darkness, sampling occurred using green-filtered head torches.

We wished to ensure that the abundance of transcripts could be compared between each sampling season. We normalized all transcript measurements to the transcript levels in one sample. Therefore, we obtained this reference sample for normalization of all RT-qPCR experiments in the study by pooling RNA from 10 leaves sampled at noon on 26 March 2015, from 10 healthy plants across the field site that were each separated by at least 1 metre. This provided a reference cDNA sample against which all RT-qPCR analyses from all sampling seasons were normalized within the ΔΔCt method (10), to allow comparability between all datasets. The reference sample was collected at midday because all transcripts under investigation were expressed to some extent at that time point. In all experiments, dawn and dusk were defined as the astronomical (solar) time of sunrise and sunset.

*RNA isolation and RT-qPCR*

Frozen samples containing RNA*later* were defrosted in a cold room for 4 hours, the RNA*later* was removed, and leaf tissue was transferred to new dry tubes and frozen in liquid nitrogen. Frozen tissue was ground with a TissueLyzer (Qiagen, Hilden, Germany) and total RNA was isolated from the powdered plant material using Macherey-Nagel Nucleospin II RNA extraction kits (Thermo Fisher Scientific). RNA concentrations were determined using a Nanodrop spectrophotometer (Thermo Fisher Scientific). cDNA was synthesized using a High Capacity cDNA Reverse Transcription Kit (Thermo Fisher Scientific) and random primers supplemented with RNAase inhibitor (Thermo Fisher Scientific), as described previously (11, 12). 1:500 cDNA dilutions were analyzed using Brilliant III Ultra-Fast SYBR Green QPCR Master Mix (Agilent Technologies, Santa Clara, CA, USA), required primer pairs (Table S2), and Agilent Mx3005P

qPCR instrument. Primers were designed using the PrimerQuest™ Tool (Integrated DNA Technologies, Coralville, IA, USA). Results were normalized using the ΔΔCt method to *AhgACTIN2* (11, 12). *AhgACTIN2* is encoded in *A. halleri* by locus *g21632* (5) and has 97.8% coding sequence identity with *A. thaliana ACTIN2* (*At3g18780*). We selected *AhgACTIN2* as a reference transcript, because it has been used previously as a reference transcript for experiments involving *A. halleri* at this field site (2), *AhgACTIN2* does not fluctuate across the seasonal cycle (1), and in our experiments, the RT-qPCR Ct for *AhgACTIN2* did not oscillate across the diel cycle (*AhgACTIN2* time-series arrhythmic after analysis with JTK_CYCLE test for rhythmicity (13); *SI Appendix*, Fig. S22). In time-series of observed data and model predictions, JTK_CYCLE was used to test for rhythmicity and estimate the phase, with phase expressed as the circular mean (calculated using the *circular* R package). Note that JTK_CYCLE reports the phase as an integer value.

*Environmental monitoring*

The temperature and irradiance were measured beside the plants during sampling. The temperature at each location, for each temperature manipulation, was monitored with EL-USB-2 data loggers (Lascar Electronics, Whiteparish, UK) at 5-minute intervals. Temperature loggers were wrapped in aluminium foil to prevent surface heating by solar radiation. Irradiance was measured using a CC-3-UV-S cosine corrector connected to a USB2000+ spectrometer with a QP400-2-UV-VIS fibre optic cable (Ocean Optics, Dunedin, FL, USA). Ambient light spectra (200 nm to 900 nm) were collected every 5 minutes over the 14 hours of light during each day of sampling using OceanView software (Ocean Optics) on a laptop PC, controlled by a custom script. The spectrometer and computer were powered using portable lithium battery packs (Powertraveller, Hampshire, UK).

*Smooth trend model*

The smooth trend model (STM) allows inference of a trend within time-series data that contains both sampling noise and biological stochasticity, and also allows a level of statistical confidence

to be applied to that trend. The STM to assess the difference in transcript abundance between March and September under sun and shade conditions (Fig. 2) was defined by the equations:

$$\mu_{1,t} \sim Normal\left(2\mu_{1,t-1} - \mu_{1,t-2},\ \sigma_{\mu 1}^2\right) \qquad (3 \leq t \leq 13), \tag{1}$$

$$\delta_t \sim Cauchy(\delta_{t-1},\ \sigma_\delta^2) \qquad (2 \leq t \leq 13), \tag{2}$$

$$\mu_{2,t} = \mu_{1,t} + \delta_t, \tag{3}$$

$$y_{1,t} \sim Normal(\mu_{1,t},\ \sigma_y^2) \qquad (1 \leq t \leq 13), \tag{4}$$

$$y_{2,t} \sim Normal(\mu_{2,t},\ \sigma_y^2) \qquad (1 \leq t \leq 13), \tag{5}$$

where $\mu_1$ and $\mu_2$ are the smooth trend components in March and September in 2015, respectively, $\delta$ is the time-varying difference between the two seasons, $y_1$ and $y_2$ are the observed transcript abundance in the two seasons, and $\sigma^2$ is the variance. $t = (1, 2, \cdots, 13)$ is the time point at two-hour intervals. The same STM was used to analyze the difference in transcript abundance between sun and shade conditions in March and September (*SI Appendix*, Fig. S9).

For the models of the three (ambient, warm and cool) conditions in the temperature manipulation experiment (Fig. 4), additional $\delta$, $\mu$ and $y$ were considered:

$$\delta_{2,t} \sim Cauchy(\delta_{2,t-1},\ \sigma_{\delta 2}^2) \qquad (2 \leq t \leq 13), \tag{6}$$

$$\mu_{3,t} = \mu_{1,t} + \delta_{2,t}, \tag{7}$$

$$y_{3,t} \sim Normal\left(\mu_{3,t},\ \sigma_y^2\right) \qquad (1 \leq t \leq 13). \tag{8}$$

The parameters of the models were estimated by Bayesian inference using the Markov Chain Monte Carlo (MCMC) approach. We note that unlike classical hypothesis testing methods, multiple comparisons do not raise a problem in a Bayesian multilevel modelling (14). The

statistical models were written in the Stan language and the programs were compiled using CmdStan (v2.24). To operate CmdStan, the cmdstanr package (v0.4.0) of R was used. After 1,000 warm-up steps, 1,000 MCMC samples were obtained by thinning out 3,000 MCMC samples for each of four parallel chains. Thus, 4,000 MCMC samples were obtained in total. We confirmed the convergence of MCMC sampling (*SI Appendix*, Figs. S5–S7). For $\mu_{1,1}$, $\mu_{1,2}$, $\delta_1$, and $\delta_{2,1}$, a flat prior over the entirety of real numbers (from negative infinity to infinity) was used to minimize prior influence on the posterior distributions. For $\sigma$, a flat prior over the range from zero to infinity was used, reflecting the underlying assumption that standard deviations cannot logically take negative values. A noninformative uniform prior is the first choice for standard deviations in hierarchical models because it does not constrain posterior inference (15), and it is also used in state-space models (16, 17). We assumed that $\sigma_y$ (observation error) is common among $y_1$, $y_2$, and $y_3$, because it is mainly derived from technical errors during RT-qPCR which is assumed to be common among different samples. We confirmed the convergence of MCMC sampling for $\sigma_y$ (*SI Appendix*, Figs. S5–S7). A cauchy distribution was used to represent the time-varying difference between two time series ($\delta$) because this distribution corrects better for the influence of outliers, due to its relatively long tails and its efficiency for detecting change points in time series data (18, 19).

*Residual analysis for smooth trend model*

Residuals were calculated as the difference between the mean observed values and predictions of the smooth trend model (STM), to assess the suitability of the model (*SI Appendix*, Fig. S8). Quantile-Quantile plots of the residuals were drawn to compare its distribution to the normal distribution, using the qqplotr package (v0.0.5) of R.

*Local level model with exogenous variables*

The local level model with exogenous variables (LLMX) to analyze a diel trend and the effect of environmental variables on transcript abundance (Fig. 3) was defined by the equations:

$$\mu_t \sim Normal(\mu_{t-1},\ \sigma_\mu^2) \qquad (2 \le t \le 13), \tag{9}$$

$$\alpha_{MarSun,t} = \mu_t + \beta_{temp} \cdot temp_{MarSun,t} + \beta_{irrad} \cdot irrad_{MarSun,t} + \beta_{gene} \cdot gene_{MarSun,t}, \tag{10}$$

$$\alpha_{MarShade,t} = \mu_t + \beta_{temp} \cdot temp_{MarShade,t} + \beta_{irrad} \cdot irrad_{MarShade,t} + \beta_{gene} \cdot gene_{MarShade,t}, \tag{11}$$

$$\alpha_{SepSun,t} = \mu_t + \beta_{temp} \cdot temp_{SepSun,t} + \beta_{irrad} \cdot irrad_{SepSun,t} + \beta_{gene} \cdot gene_{SepSun,t}, \tag{12}$$

$$\alpha_{SepShade,t} = \mu_t + \beta_{temp} \cdot temp_{SepShade,t} + \beta_{irrad} \cdot irrad_{SepShade,t} + \beta_{gene} \cdot gene_{SepShade,t}, \tag{13}$$

$$y_{MarSun,t} \sim Normal(\alpha_{MarSun,t},\ \sigma_y^2) \qquad (1 \le t \le 13), \tag{14}$$

$$y_{MarShade,t} \sim Normal(\alpha_{MarShade,t},\ \sigma_y^2) \qquad (1 \le t \le 13), \tag{15}$$

$$y_{SepSun,t} \sim Normal(\alpha_{SepSun,t},\ \sigma_y^2) \qquad (1 \le t \le 13), \tag{16}$$

$$y_{SepShade,t} \sim Normal(\alpha_{SepShade,t},\ \sigma_y^2) \qquad (1 \le t \le 13), \tag{17}$$

where $\mu$ is the autoregressive trend component that is common among conditions, $\beta$ is the regression coefficient, $\alpha$ is the true state of transcript abundance, $y$ is the observed transcript abundance, and $\sigma^2$ is the variance. The subscripts, $temp$, $irrad$, $gene$, $Mar$, $Sep$, $Sun$ and $Shade$ represent temperature, irradiance, an upstream gene, March, September, sun condition and shade condition, respectively. The mean transcript abundance of the upstream gene (i.e., *AhgCCA1* in the *AhgSIG5* model and *AhgSIG5* in the *AhgpsbD* BLRP model) was used as one of the inputs. $t = (1, 2, \cdots, 13)$ is the time point at two-hour intervals.

The parameters of the models were estimated by Bayesian inference using the Markov Chain Monte Carlo (MCMC) approach (Table S2, S3). The statistical models were written in the Stan language and the programs were compiled using CmdStan (v2.24). To operate CmdStan, the cmdstanr package (v0.4.0) of R was used. After 3,000 warm-up steps, 1,000 MCMC samples were obtained for each of the four parallel chains, and thus 4,000 MCMC samples were obtained

in total. We confirmed the convergence of MCMC sampling (*SI Appendix*, Figs. S10 and S12). For $\beta$ and $\mu_1$, a flat prior over the entire real numbers (from negative infinity to infinity) was used to minimize prior influence on the posterior distributions. For $\sigma$, a flat prior over the range from zero to infinity was used, reflecting the underlying assumption that standard deviations cannot logically take negative values. A noninformative uniform prior is the first choice for standard deviations in hierarchical models because it does not constrain posterior inference (15), and it is also used in state-space models (16, 17). We assumed that $\sigma_y$ (observation error) is common among $y_{MarSun}$, $y_{MarShade}$, $y_{SepSun}$, and $y_{SepShade}$, because it is mainly derived from technical errors during RT-qPCR which is assumed to be common among different samples. We confirmed the convergence of MCMC sampling for $\sigma_y$ (*SI Appendix*, Figs. S10 and S12).

*Embedding dimension*

In the empirical dynamic modelling (EDM), time series is embedded into time-lagged series, a procedure known as state space reconstruction (20, 21). The embedding dimension *E* is the dimension (i.e. the number of time-lagged series) used to reconstruct the state space. Prior to convergent cross mapping (CCM), the optimal *E* value was determined for each of *AhgCCA1*, *AhgSIG5* and *AhgpsbD* BLRP in temperature manipulation experiments in September 2016, by univariate simplex projection (22), using the simplex function of the rEDM package (v0.7.5) of R. We determined the optimal *E* values showing the maximum forecast skill ρ (*SI Appendix*, Fig. S17A). The optimal *E* value of 8 for *AhgCCA1* was relatively large, considering those of *AhgSIG5* and *AhgpsbD* BLRP, and data points available in this analysis. We adopted *E* value of 2 as the optimal value for *AhgCCA1*, because ρ is higher than 0.9 for *E* values from 2 to 8 (*SI Appendix*, Fig. S17A) and the model prediction well fits the observed data at *E* value of 2 (*SI Appendix*, Fig. S17B). The optimal *E* values determined were 2 for *AhgCCA1*, 4 for *AhgSIG5*, and 2 for *AhgpsbD* BLRP. The model predictions well fit the observed data for these genes at the determined *E* values (*SI Appendix*, Fig. S17B-D).

*Convergent cross mapping*

When a causal relationship exists from a variable *X* to another variable *Y*, the information of *X* can be found in the time series of *Y*, meaning that the prediction of *X* is possible using the information of *Y*. In convergent cross mapping (CCM) based on simplex projection, one predicts the nearest neighbors of *X* at time *t* in the reconstructed lagged trajectory, using their time-corresponding points of *Y* (23). When this prediction (called cross mapping) is successful, the causality from *X* to *Y* is assumed. The cross map skill (i.e. prediction skill) is evaluated by Pearson's correlation coefficient (ρ) between predicted and observed values. We performed CCM between *AhgCCA1* (*X*) and *AhgSIG5* (*Y*), and between *AhgSIG5* (*X*) and *AhgpsbD* BLRP (*Y*) for the temperature manipulation experiments (September 2016), using the ccm function in the rEDM package (v0.7.5) of R. We considered time delay in the interactions by changing the parameter tp from −6 to 2 (negative, past; positive, future) in the ccm function. When causality exists, optimal predictability is expected to occur for tp ≤ 0, i.e, prediction of past values of *X* from *Y* (24). We used a technique known as multispatial CCM (25), to utilize the relatively small number of time points of 13/condition in 2015 and 15/condition in 2016. For the 2015 data, we used two light conditions (sun and shade) for each of March and September, giving a total of 26 time points applied to CCM. For the 2016 data, we used three temperature conditions (ambient, warm and cool), giving a total of 45 time points applied to CCM. To test the significance of cross map skill, we produced 1,000 diel surrogate time series for *X* into which a similar level of oscillation was incorporated, but the sequence of data deviation from the oscillation was randomized. We used three criteria for significant causality. First, optimal predictability occurs for tp ≤ 0, second, cross map skill is above the 95 % interval of diel surrogates at the optimal time lag (tp), third, cross map skill is improved according to the increase in a library size (number of time points used to reconstruct a state space), known as convergence (23).

*Application of the LLMX model to the RNA-seq data*

We used reanalyzed RNA-seq count data from Nishio et al. (2020) (26), with sequences originally obtained from four sets of 48-hour samples taken at 2-hour intervals in 2013 (Nagano et al., 2019

*Nat. Plants*) (1). As environmental variables, we used ambient temperature at 2-hour intervals and hourly accumulated solar radiation recorded at the meteorological station in Osaka, the closest station to our field site with both environmental variables available. The same LLMX model (equations 9-17) was applied to each signalling pathway. For the modelling of *AhgCOR15A* (*SI Appendix*, Fig. S21), an additional term of a gene was added to equations 10-13. The parameters of the models were estimated by Bayesian inference as described in the previous section.

**Data and code availability**

Source data are provided in Datasets S1. Crucial code for modelling is provided at https://github.com/hnishio/SIG5_field_PNAS.git.

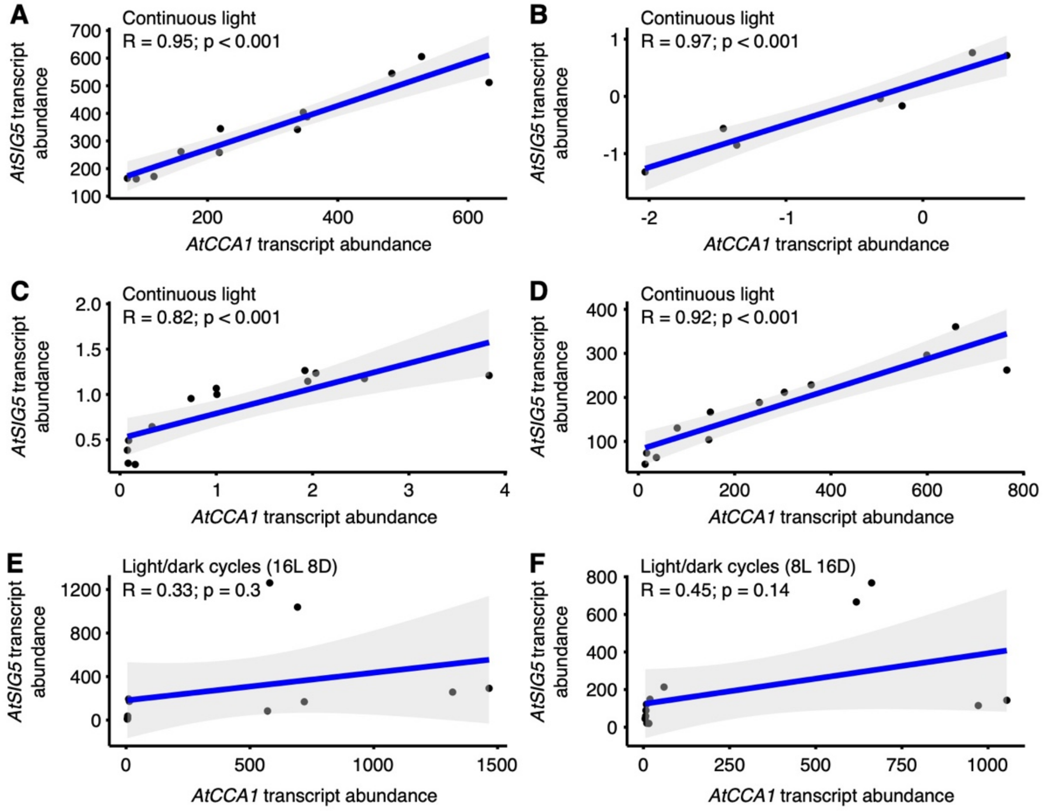


**Fig. S1.** Relationship between *AtCCA1* and *AtSIG5* transcript abundance in *A. thaliana* under controlled conditions. (A-D) Relationship between *AtCCA1* and *AtSIG5* transcript abundance under conditions of constant light, from the transcriptome studies of (A) (27) (B) (28), (C) (29), (D) (30). (E, F) Relationship between *AtCCA1* and *AtSIG5* transcript abundance under light/dark cycles with (E) long and (F) short photoperiods, from the transcriptome study of (27, 31). Blue lines indicate a regression line. Pearson's correlation coefficient (R) with the corresponding p-values, testing the likelihood of a chance correlation, are shown for each plot.

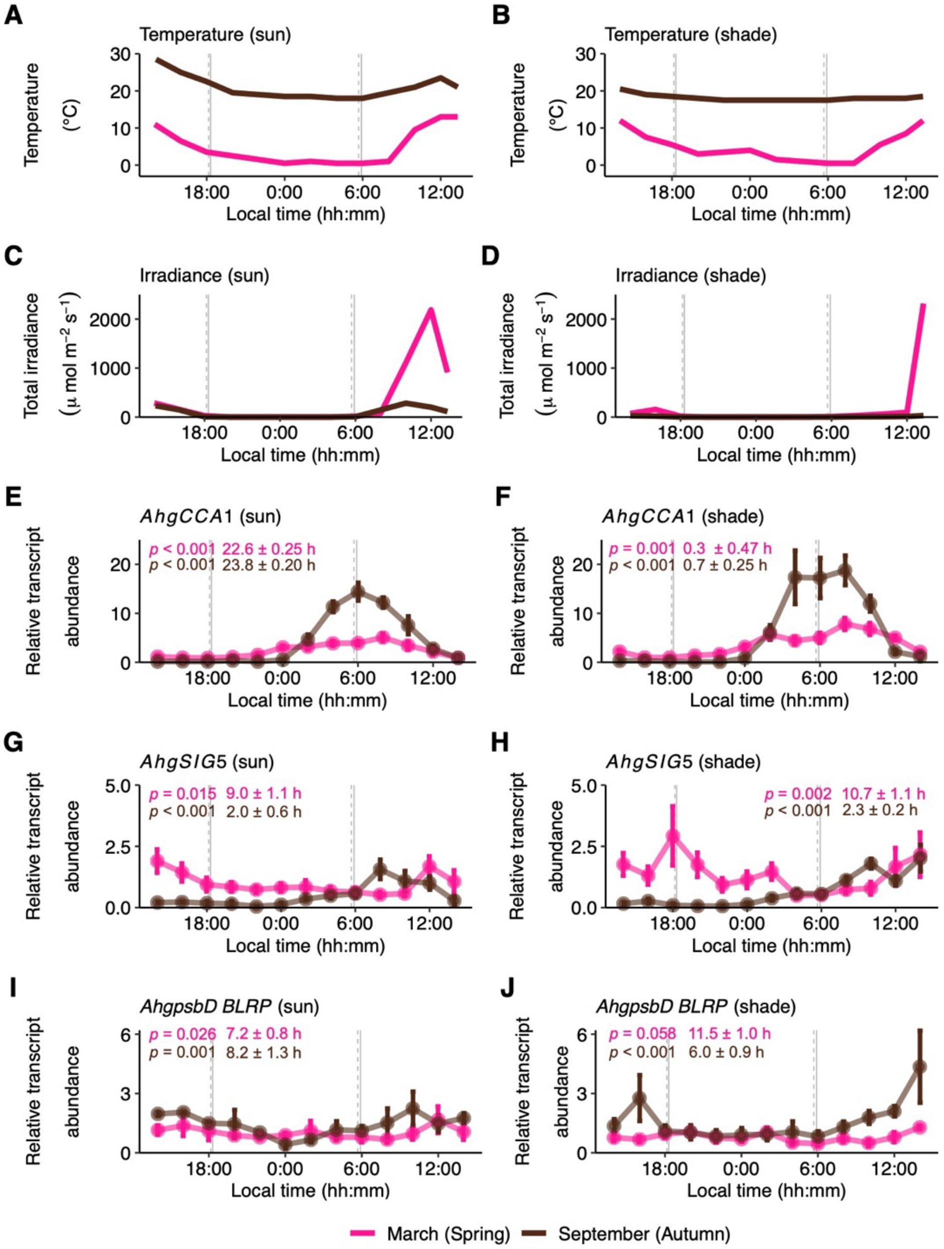


**Fig. S2.** Data underlying models produced in this study, here comparing signalling pathway dynamics between two seasons, under different light conditions in a natural population of *A. halleri*. (A-D) Diel fluctuations in (A, B) ambient temperature and (C, D) total irradiance detected (200-900 nm), at 2-hour intervals (thinned out from original data measured at 5-minute intervals, for the purpose of aligning intervals with the transcript data) during sampling period in March and September 2015. (E-J) Transcript abundance of (E, F) *AhgCCA1*, (G, H) *AhgSIG5* and (**I**, J) *AhgpsbD BLRP*. Vertical grey lines on time-series plots indicate the times of sunrise and sunset during March (solid line) and September (dashed). Panels E-J include estimation of rhythmicity and peak time relative to solar dawn of underlying data, using JTK_CYCLE. Data are mean ± s.e.m; $n$ = 6 replicate plants.

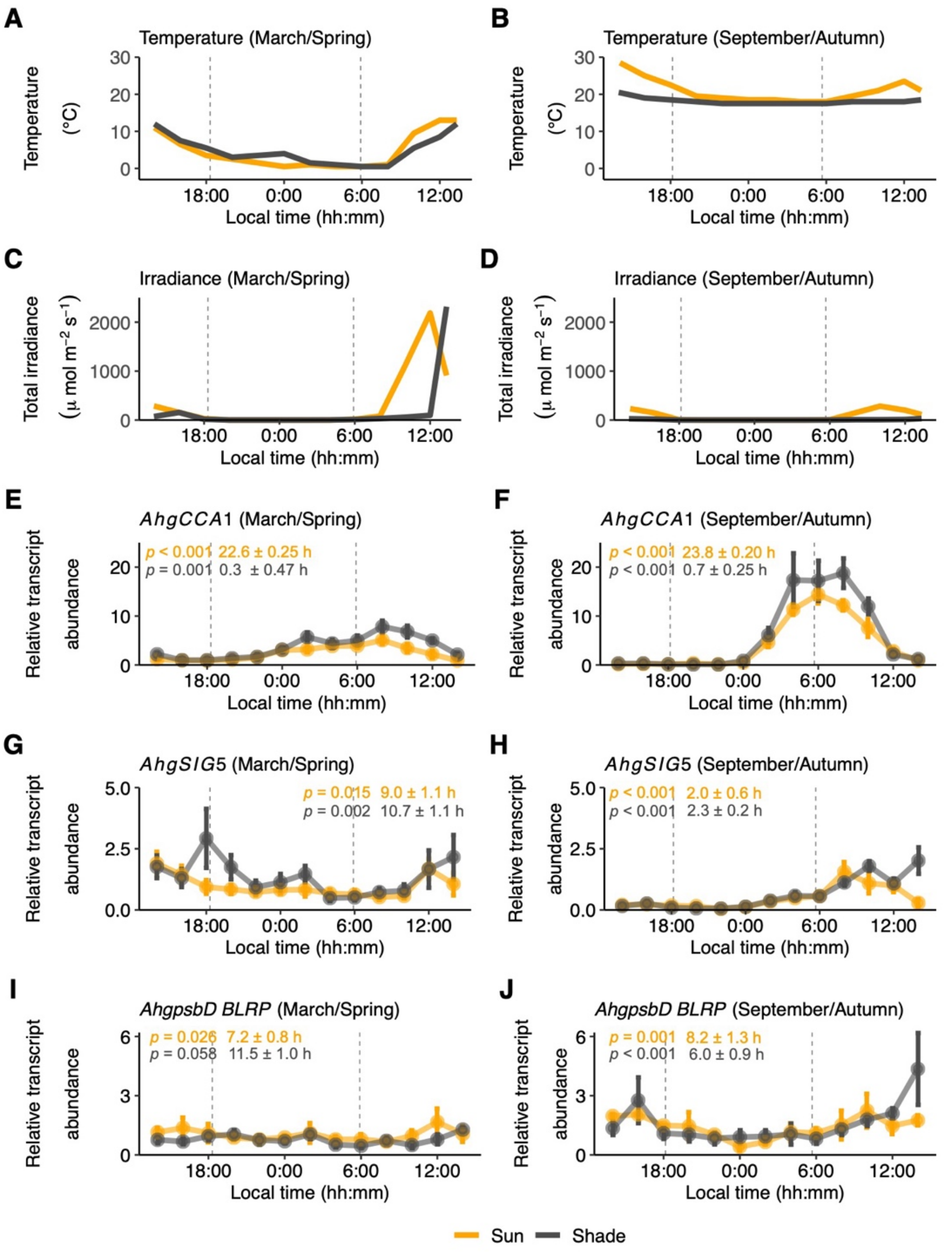


**Fig. S3.** Data underlying models produced in this study, here comparing signalling pathway dynamics between two different light conditions, during two sampling seasons in a natural population of *A. halleri*. (A-D) Diel fluctuations in (A, B) ambient temperature and (C, D) total irradiance detected (200-900 nm), at 2-hour intervals (thinned out from original data measured at 5-minute intervals, for the purpose of aligning intervals with the transcript data) during sampling period in March and September 2015. (E-J) Transcript abundance of (E, F) *AhgCCA1*, (G, H) *AhgSIG5* and (I, J) *AhgpsbD BLRP*. Panels E-J include estimation of rhythmicity and peak time relative to solar dawn of underlying data, using JTK_CYCLE. Vertical grey lines on time-series plots indicate the times of sunrise and sunset. Data are mean ± s.e.m; $n$ = 6 replicate plants.

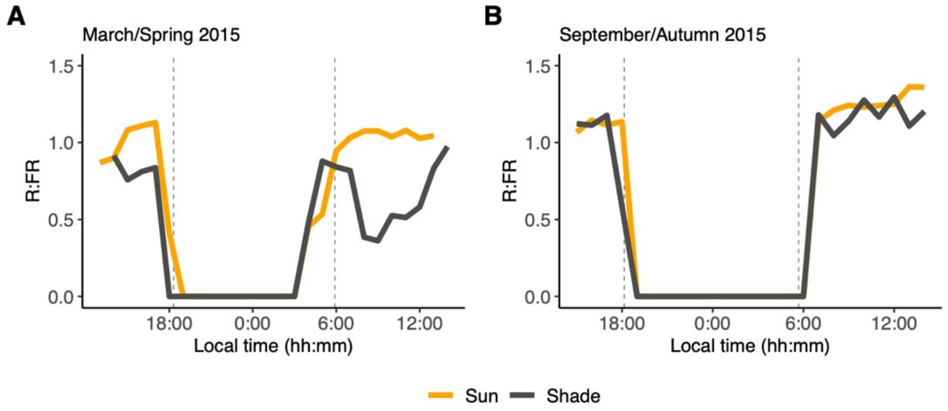


**Fig. S4.** The ratio of red to far-red light in a natural population of *A. halleri*, during March and September sampling seasons. (A, B) Comparison of the ratio of red to far-red light received by plants under the sun- and shade conditions during (A) March 2015 and (B) September 2015 sampling seasons. The R:FR varied during the photoperiod during both sampling seasons, and the effect of shade on R:FR was ameliorated by heavy cloud cover. Vertical grey lines on graphs indicate the times of sunrise and sunset.

**A**

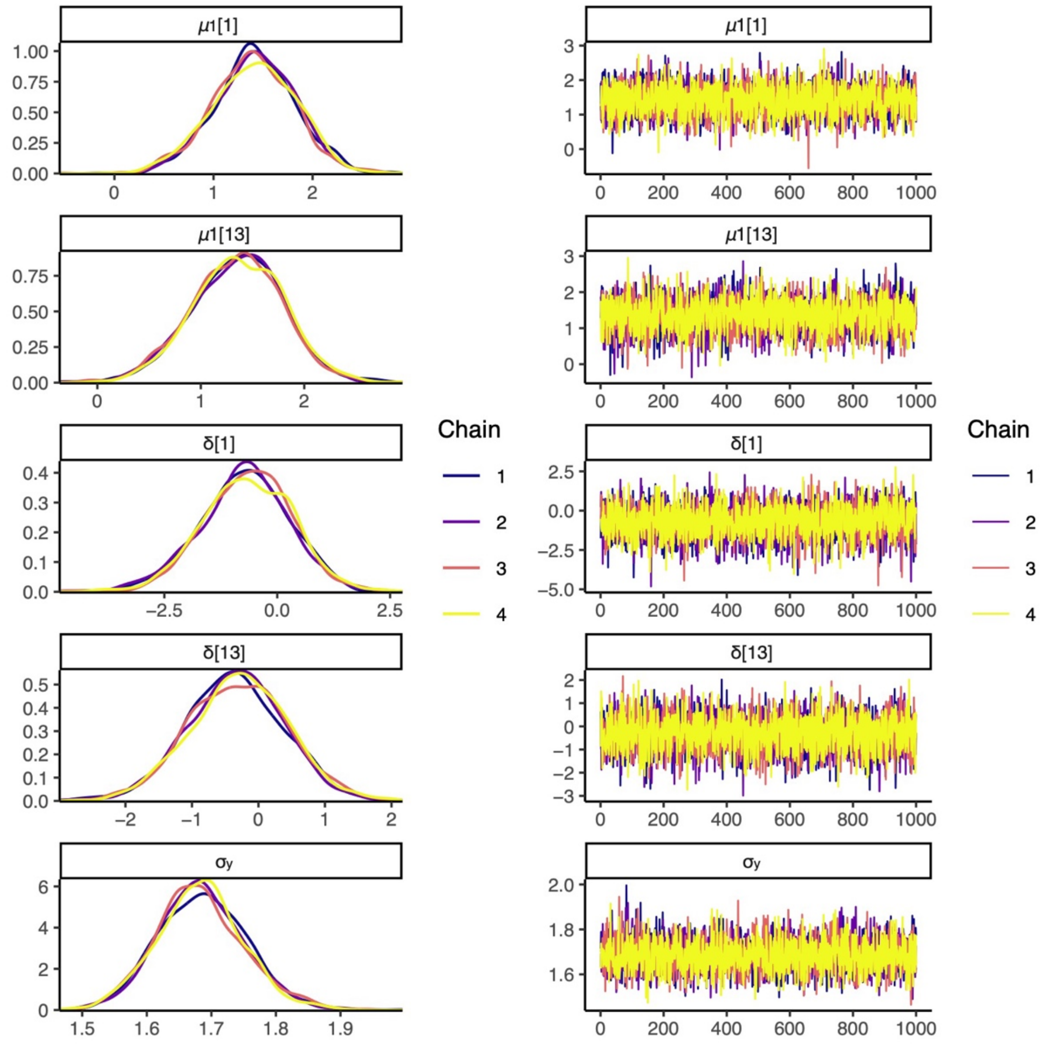


**B**

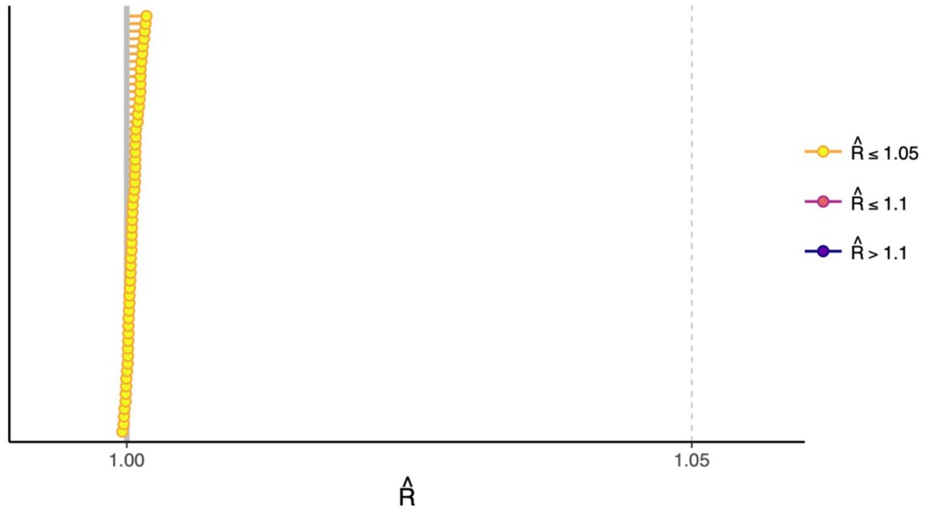


**Fig. S5.** Convergence of Markov Chain Monte Carlo (MCMC) sampling for smooth trend model (STM) for *AhgCCA1* at the sun sampling site. (A) Density plots (left) and trace plots (right) of 1,000 MCMC samples/chain generated from posterior distributions of $\boldsymbol{\mu 1}$ (a smooth trend

component), $\boldsymbol{\delta}$ (a time-varying difference between two time series), and $\boldsymbol{\sigma_y}$ (observation error as a standard deviation) after 1,000 warm-up steps. $\boldsymbol{\mu 1}[\mathbf{1}]$ and $\boldsymbol{\mu 1}[\mathbf{13}]$ represent the smooth trend component values at the first and last time points. (B) R-hat values (an indicator of how well chains are mixed, calculated by comparing within-chain variance and total variance) of all model parameters. R-hat ≤ 1.05 is recommended by the stan development team (https://mc-stan.org/rstan/reference/Rhat.html).

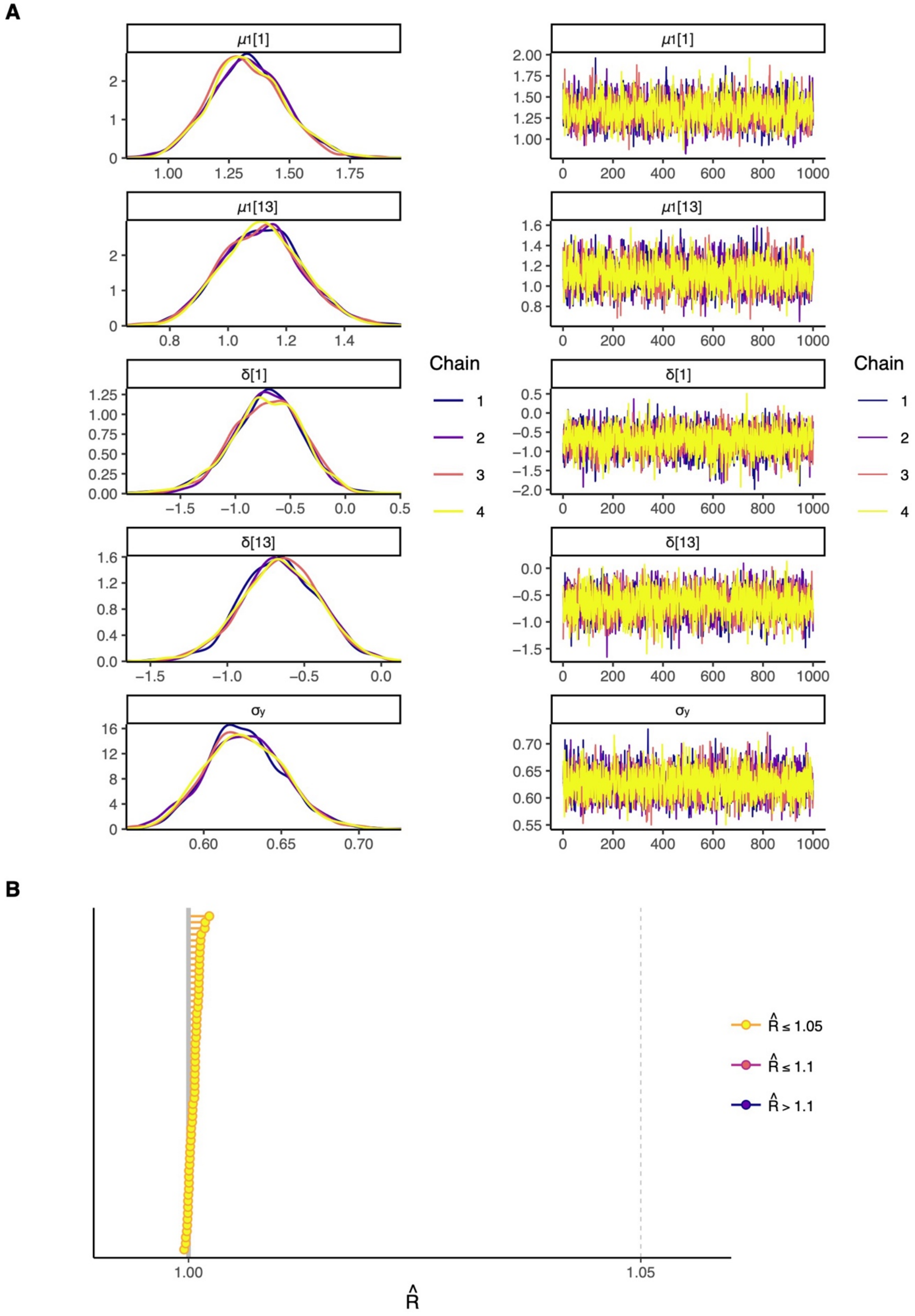


**Fig. S6.** Convergence of Markov Chain Monte Carlo (MCMC) sampling for smooth trend model (STM) for *AhgSIG5* at the sun sampling site. (A) Density plots (left) and trace plots (right) of 1,000 MCMC samples/chain generated from posterior distributions of $\boldsymbol{\mu 1}$ (a smooth trend component),

$\boldsymbol{\delta}$ (a time-varying difference between two time series), and $\boldsymbol{\sigma_y}$ (observation error as a standard deviation) after 1,000 warm-up steps. $\boldsymbol{\mu 1}[\mathbf{1}]$ and $\boldsymbol{\mu 1}[\mathbf{13}]$ represent the smooth trend component values at the first and last time points. (B) R-hat values (an indicator of how well chains are mixed, calculated by comparing within-chain variance and total variance) of all model parameters. R-hat ≤ 1.05 is recommended by the stan development team (https://mc-stan.org/rstan/reference/Rhat.html).

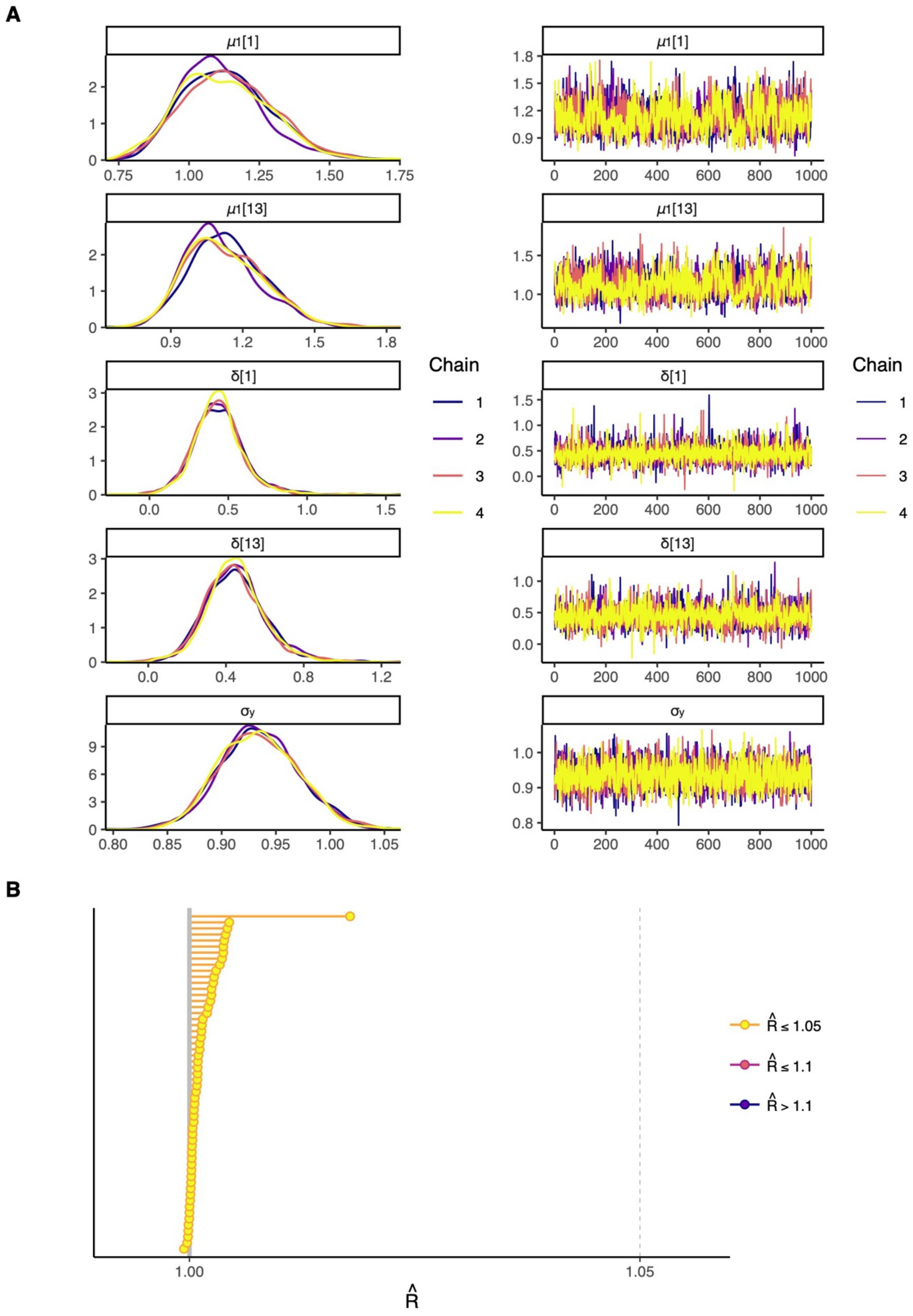


**Fig. S7.** Convergence of Markov Chain Monte Carlo (MCMC) sampling for smooth trend model (STM) for *AhgpsbD* BLRP at the sun sampling site. (A) Density plots (left) and trace plots (right) of 1,000 MCMC samples/chain generated from posterior distributions of $\boldsymbol{\mu 1}$ (a smooth trend

component), $\boldsymbol{\delta}$ (a time-varying difference between two time series), and $\boldsymbol{\sigma_y}$ (observation error as a standard deviation) after 1,000 warm-up steps. $\boldsymbol{\mu 1[1]}$ and $\boldsymbol{\mu 1[13]}$ represent the smooth trend component values at the first and last time points. (B) R-hat values (an indicator of how well chains are mixed, calculated by comparing within-chain variance and total variance) of all model parameters. R-hat ≤ 1.05 is recommended by the stan development team (https://mc-stan.org/rstan/reference/Rhat.html).

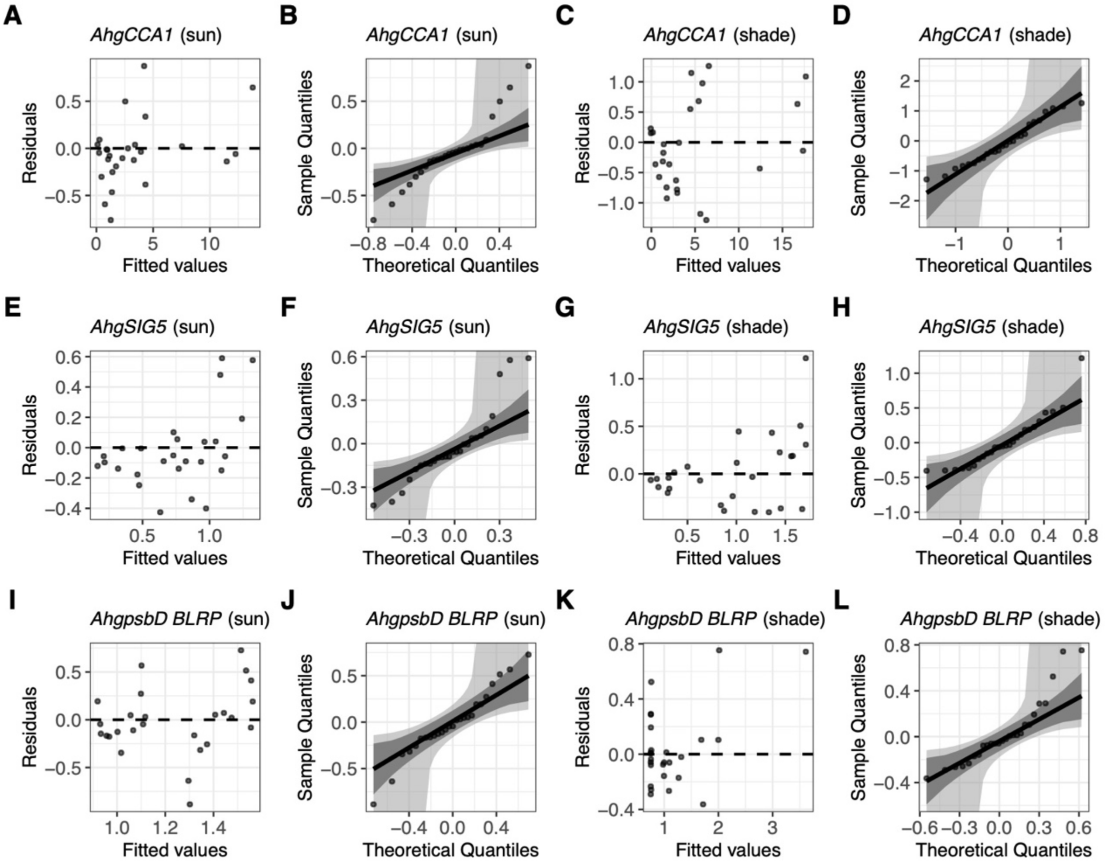


**Fig. S8.** Residual analysis of the smooth trend model (STM) for the 2015 data. (A-L) Residual plots (left) and Quantile-Quantile plots of the residuals (right) for (A, B) *AhgCCA1* (sun), (C, D) *AhgCCA1* (shade), (E, F) *AhgSIG5* (sun), (G, H) *AhgSIG5* (shade), (I, J) *AhgpsbD* BLRP (sun), and (K, L) *AhgpsbD* BLRP (shade). The thick shaded regions are pointwise confidence bands (95 %) based on normal confidence intervals. The thin shaded regions are simultaneous confidence bands (95 %) based on the Kolmogorov-Smirnov test.

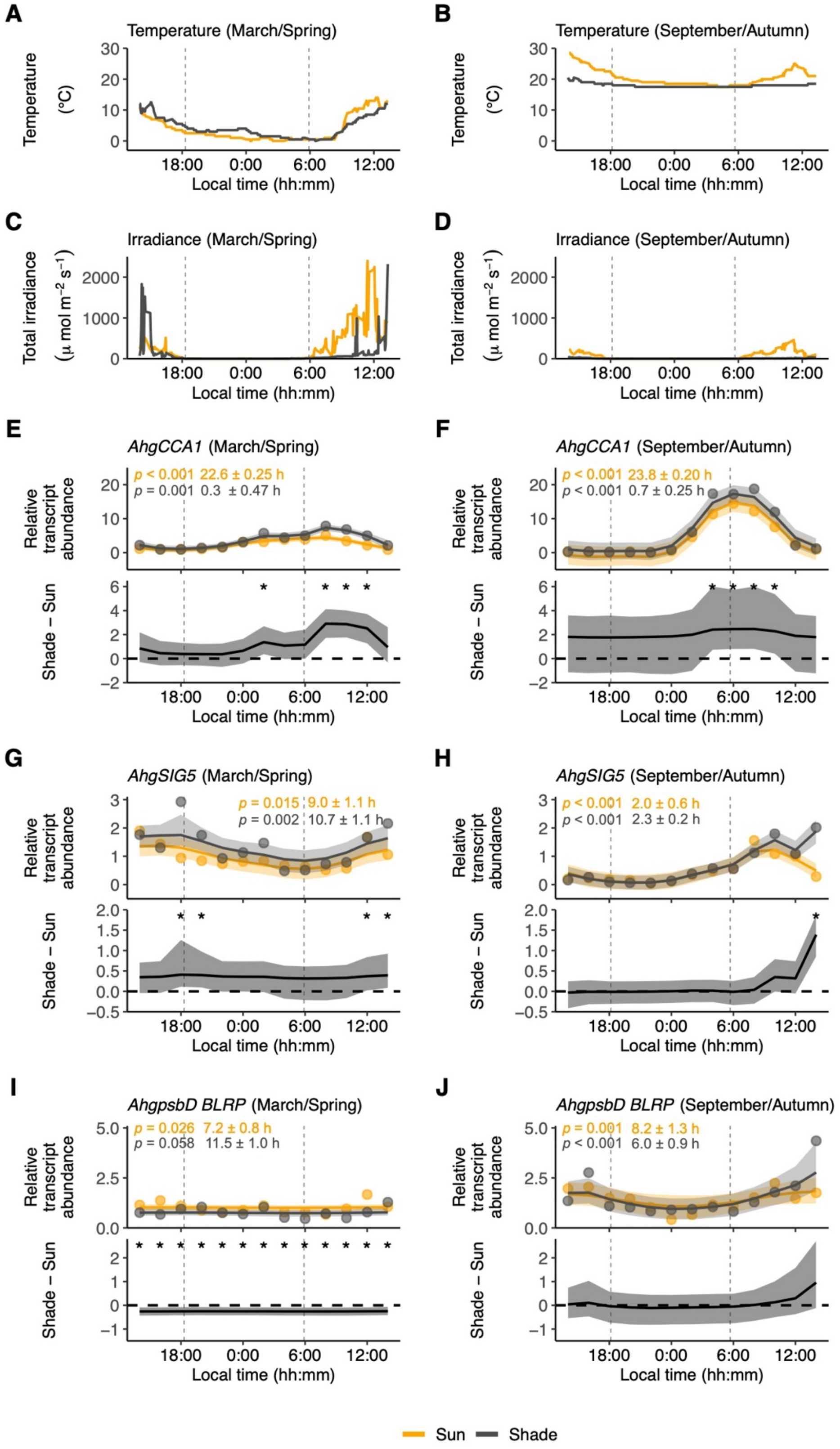


**Fig. S9.** Distinct diel dynamics of components of a circadian signalling pathway between sun and shade conditions in a natural population of *A. halleri*. (A-D) Diel fluctuations in (A, B) ambient temperature and (C, D) total irradiance detected (200-900 nm), measured at 5-minute intervals

during sampling period in March and September 2015. (E-J) Bayesian estimation of smooth trend model (STM) for transcript dynamics of (E, F) *AhgCCA1*, (G, H) *AhgSIG5* and (I, J) *AhgpsbD BLRP*. In each panel, the upper graphs show the predicted relative transcript abundance for sun (orange; $\boldsymbol{\mu_1}$, equation 1 in Materials and methods) and shade (light grey; $\boldsymbol{\mu_2}$, equation 3) conditions with the mean of observed values (dots), and the lower graphs represent the differences in transcript abundance between sun and shade conditions ($\boldsymbol{\delta}$, equation 2). The solid line and the shaded region are the median and the 95% confidence interval of the posterior distribution. When the 95% confidence interval of the difference between sun and shade conditions does not contain zero, the difference is considered significant and is indicated by asterisks. Panels E-J include estimation of rhythmicity and peak time relative to solar dawn of underlying data, using JTK_CYCLE. Vertical grey lines on time-series plots indicate the times of sunrise and sunset. STM analysis used data from 6 replicate plants per condition.

A

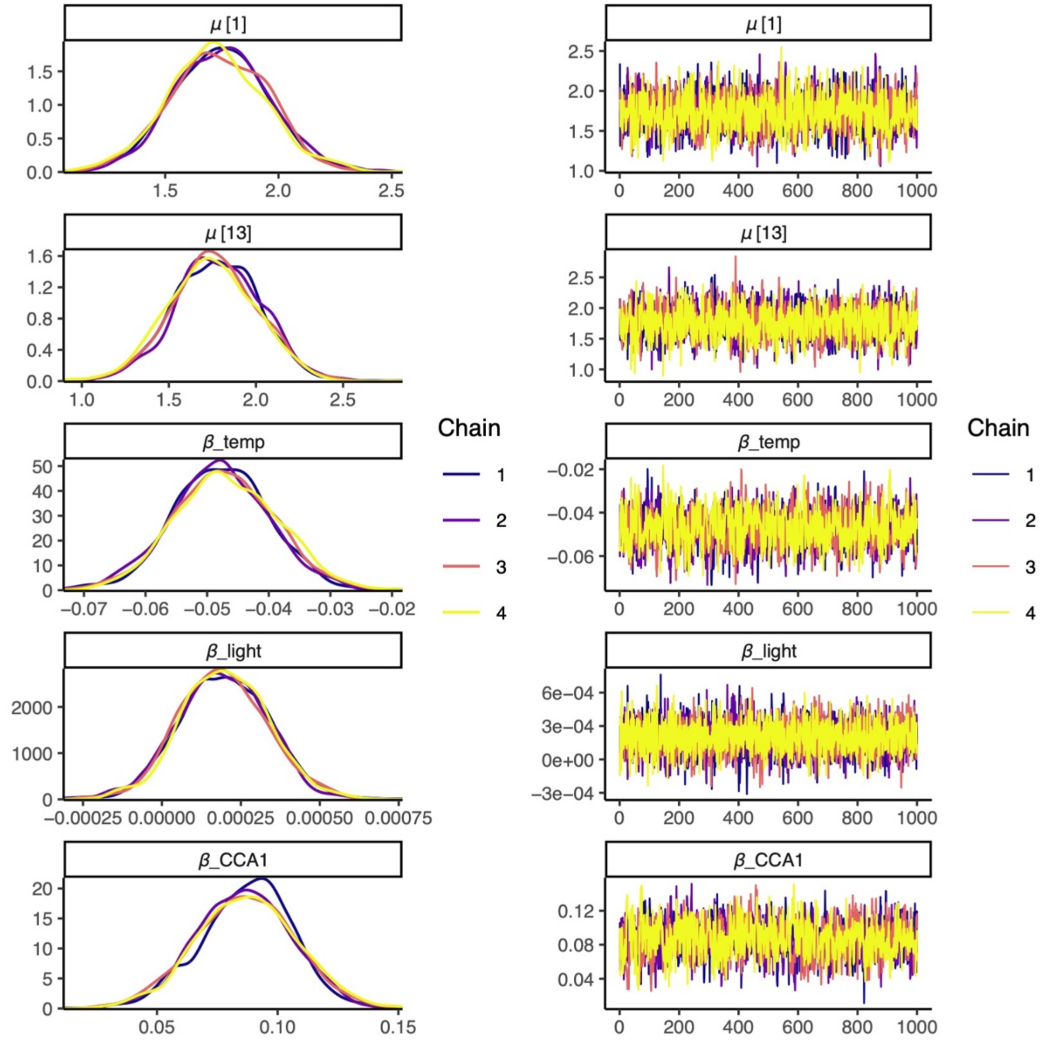


B

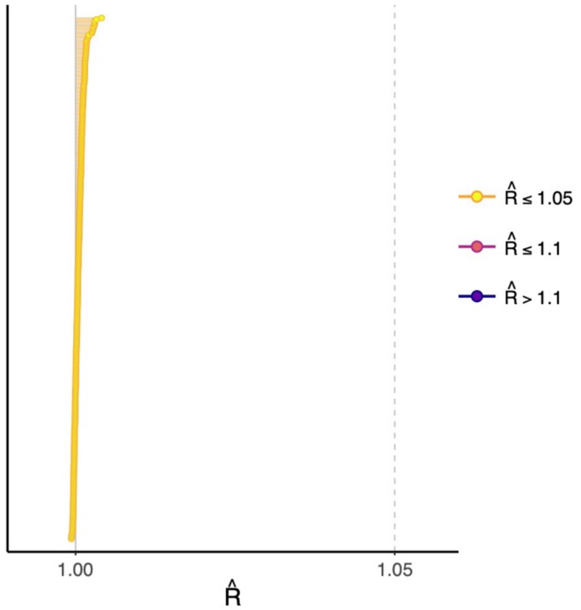


**Fig. S10.** Convergence of Markov Chain Monte Carlo (MCMC) sampling for the local level model with exogenous variables (LLMX) for *AhgSIG5*. (A) Density plots (left) and trace plots (right) of

1,000 MCMC samples/chain generated from posterior distributions of $\boldsymbol{\mu}$ (auto regressive trend component) and $\boldsymbol{\beta}$ (regression coefficients of temperature [temp], irradiance [light] and upstream gene [CCA1]), after 3,000 warm-up steps. $\boldsymbol{\mu}[\mathbf{1}]$ and $\boldsymbol{\mu}[\mathbf{13}]$ represent the trend component values at the first and last time points. (B) R-hat values (an indicator of how well chains are mixed, calculated by comparing within-chain variance and total variance) of all model parameters. R-hat ≤ 1.05 is recommended by the stan development team (https://mc-stan.org/rstan/reference/Rhat.html).

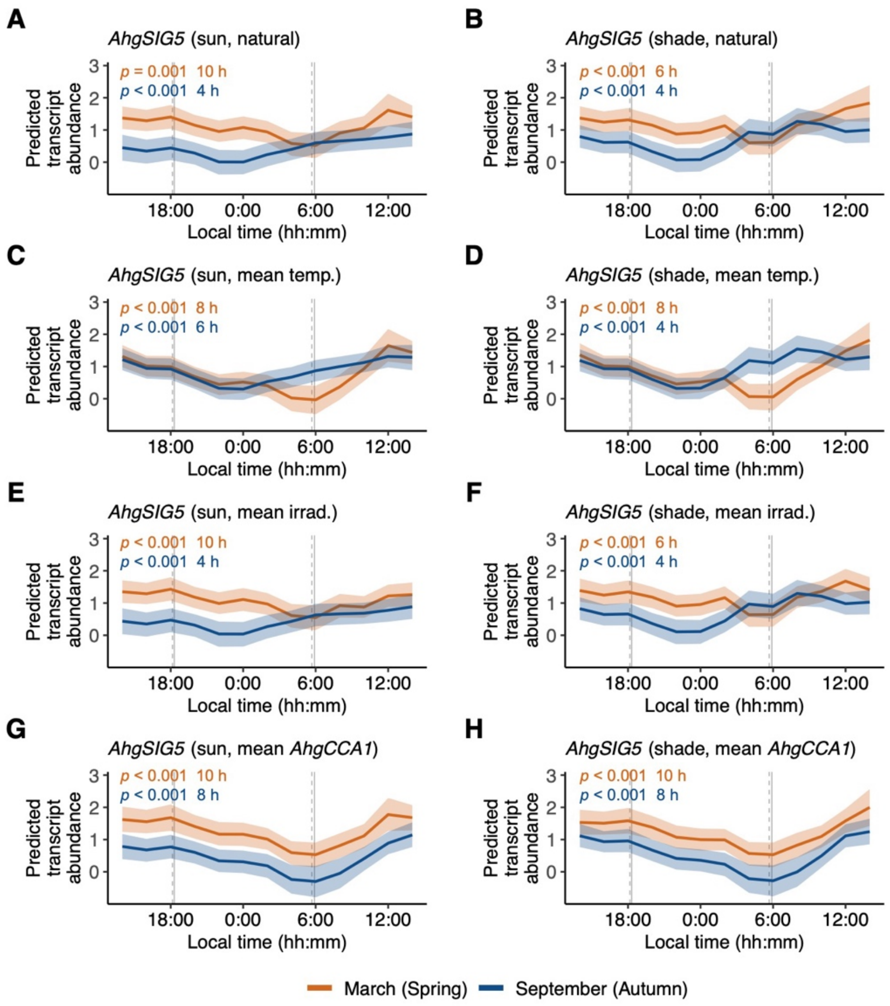


**Fig. S11.** Prediction of *AhgSIG5* transcript abundance using estimated parameter values in the local level model with exogenous variables (LLMX), when specific input variables are given as constant values. (A, B) LLMX prediction of *AhgSIG5* transcript abundance ($\boldsymbol{\alpha}$, equations 10-13 in Materials and methods) where all variables are allowed to follow natural fluctuations (as in Fig. 3A, B). (C-H) LLMX prediction of *AhgSIG5* transcript abundance ($\boldsymbol{\alpha}$) where (C, D) ambient temperature, (E, F) irradiance and (G, H) *AhgCCA1* transcript abundance were fixed at their mean value among all conditions**.** Shaded area represents 95% confidence interval. Vertical grey lines on time-series plots indicate the times of sunrise and sunset during March (solid line) and September (dashed). Panels A-H include estimation of rhythmicity and peak time relative to solar dawn of model predictions, using JTK_CYCLE.

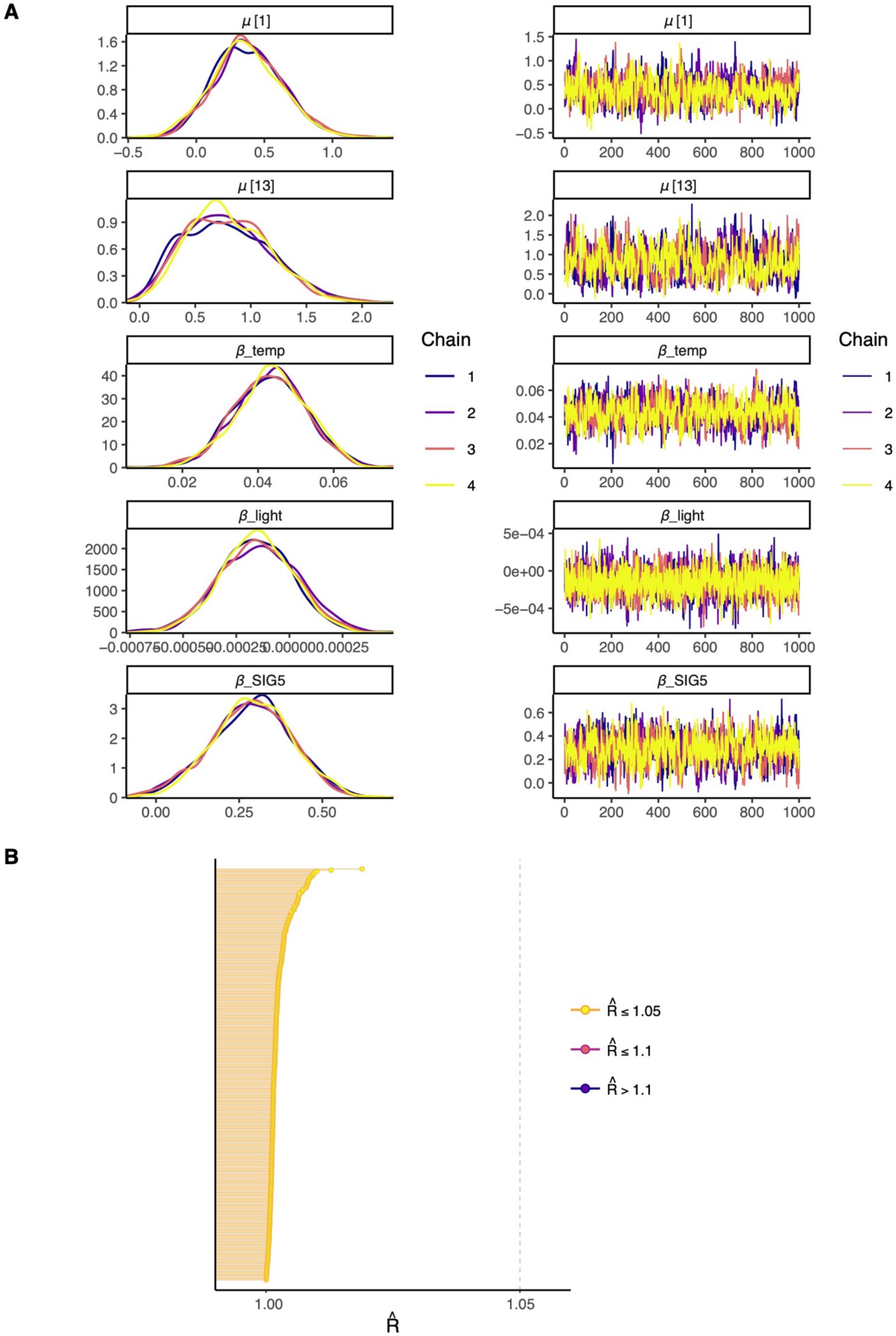


**Fig. S12.** Convergence of Markov Chain Monte Carlo (MCMC) sampling for the local level model with exogenous variables (LLMX) for *AhgpsbD* BLRP. (A) Density plots (left) and trace plots

(right) of 1,000 MCMC samples/chain generated from posterior distributions of $\boldsymbol{\mu}$ (auto regressive trend component) and $\boldsymbol{\beta}$ (regression coefficients of temperature [temp], irradiance [light] and upstream gene [SIG5]), after 3,000 warm-up steps. $\boldsymbol{\mu}[\mathbf{1}]$ and $\boldsymbol{\mu}[\mathbf{13}]$ represent the trend component values at the first and last time points. (B) R-hat values (an indicator of how well chains are mixed, calculated by comparing within-chain variance and total variance) of all model parameters. R-hat ≤ 1.05 is recommended by the stan development team (https://mc-stan.org/rstan/reference/Rhat.html).

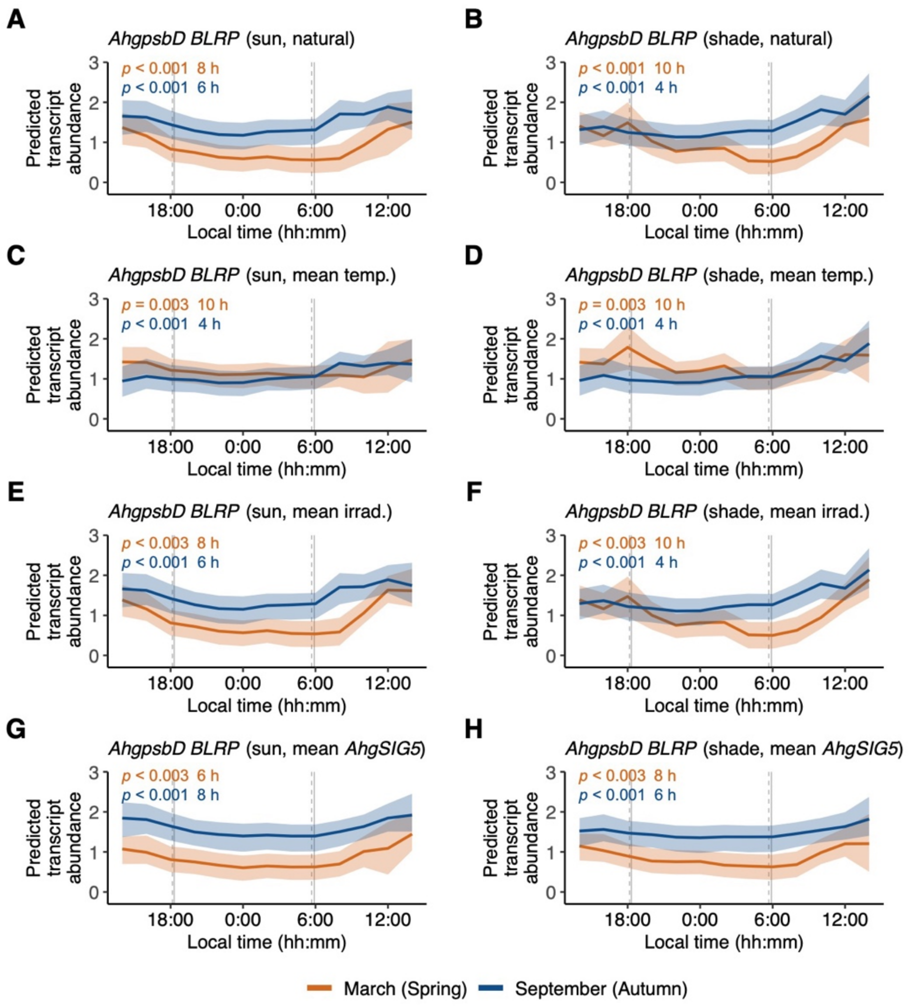


**Fig. S13.** Prediction of *AhgpsbD* BLRP transcript abundance using estimated parameter values in the local level model with exogenous variables (LLMX), when specific input variables are given as constant values. (A, B) LLMX prediction of *AhgpsbD* BLRP transcript abundance ($\boldsymbol{\alpha}$, equations 10-13 in Materials and methods) where all variables are allowed to follow natural fluctuations (as in Fig. 3E, F). (C-H) LLMX prediction of *AhgpsbD* BLRP transcript abundance ($\boldsymbol{\alpha}$) where (C, D) ambient temperature, (E, F) irradiance and (G, H) *AhgSIG5* transcript abundance were fixed at their mean value among all conditions. Shaded area represents 95% confidence interval. Vertical grey lines on time-series plots indicate the times of sunrise and sunset during March (solid line) and September (dashed). Panels A-H include estimation of rhythmicity and peak time relative to solar dawn of model predictions, using JTK_CYCLE.

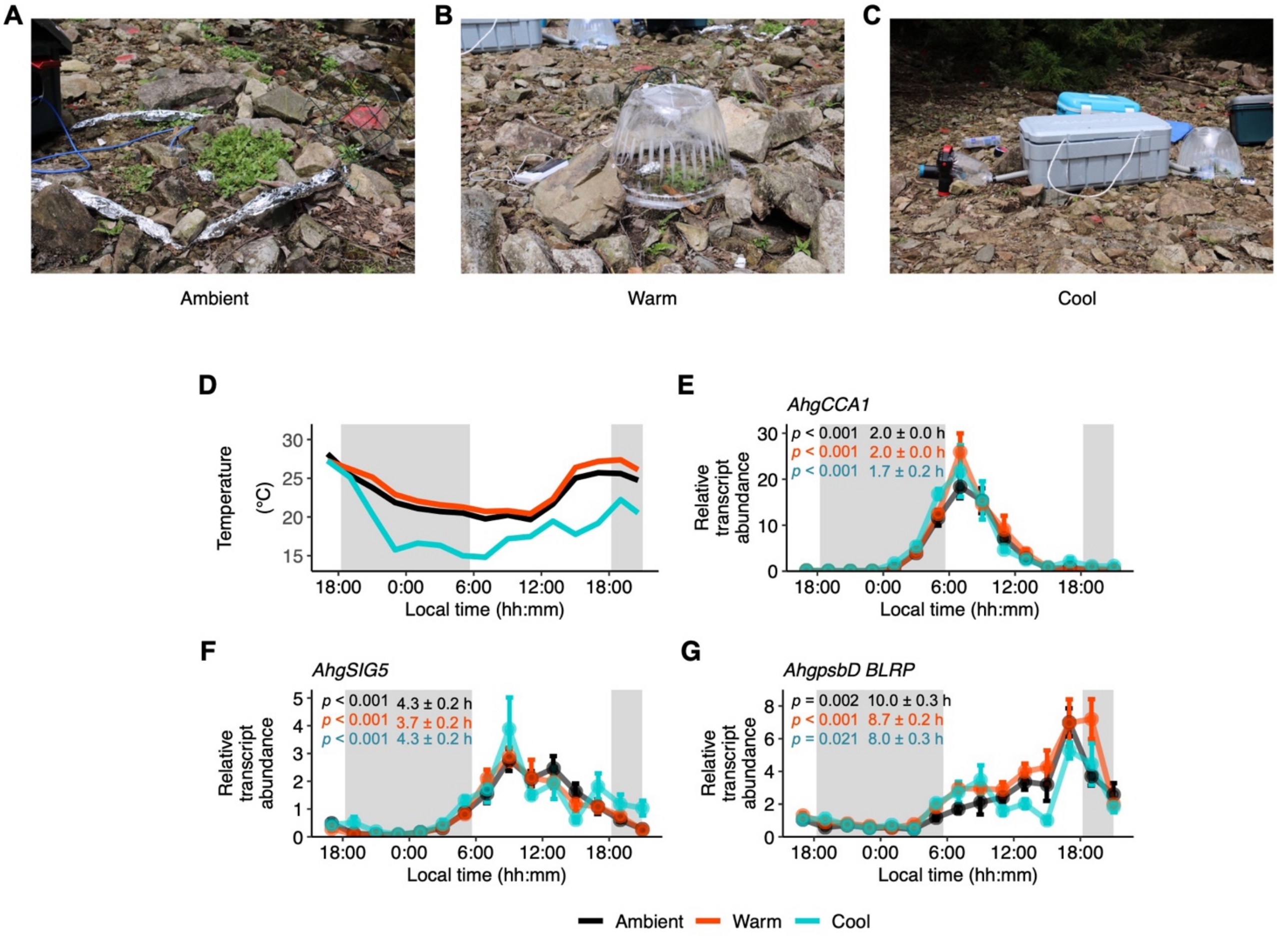


**Fig. S14.** Moderate temperature manipulations applied to patches of *A. halleri* plants, in the field, using custom-designed equipment. (A) Representative appearance of plant patches under naturally fluctuating conditions. (B) Plants covered with a plastic dome to raise the temperature. (C) Plants covered with a plastic dome undergoing temperature reduction with a custom chilling device. In this device, cool air is introduced to enclosed plant patches after being driven slowly through a heat exchanger, positioned within an expanded polystyrene box filled with ice. (D) Temperature changes in each condition at 2-hour intervals (thinned out from original data measured at 5-minute intervals, for the purpose of aligning intervals with the transcript data) during sampling period in September 2016. (E-G) Fluctuations in *AhgCCA1*, (F) *AhgSIG5* and (G) *AhgpsbD* BLRP transcript abundance under ambient conditions and following temperature manipulation of plant patches. Panels E-G include estimation of rhythmicity and peak time relative to solar dawn of underlying data, using JTK_CYCLE. Grey shaded boxes on graphs indicate the period between sunset and sunrise. Data are mean ± s.e.m; $n$ = 6 replicate plants.

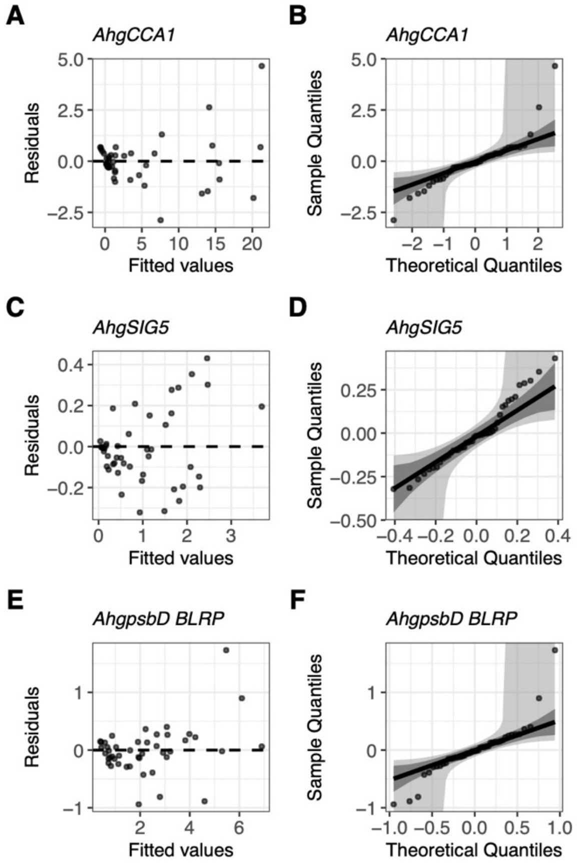


**Fig. S15.** Residual analysis of the smooth trend model (STM) for temperature manipulation experiments in September 2016. (A-F) Residual plots (left) and Quantile-Quantile plots of the residuals (right) for (A, B) *AhgCCA1*, (C, D) *AhgSIG5*, (E, F) *AhgpsbD* BLRP. The thick shaded regions are pointwise confidence bands (95 %) based on normal confidence intervals. The thin shaded regions are simultaneous confidence bands (95 %) based on the Kolmogorov-Smirnov test.

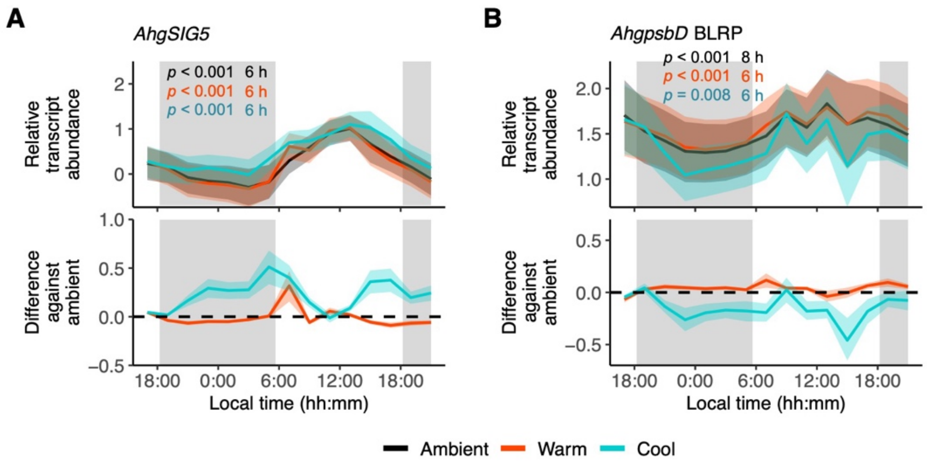


**Fig. S16.** Prediction of the transcript abundance of *AhgSIG5* and *AhgpsbD* BLRP in the 2016 experiment, using the local level model with exogenous variables (LLMX) in which parameter values are estimated from the 2015 data. (A, B) Predicted transcript dynamics of (A) *AhgSIG5* and (B) *AhgpsbD BLRP*. In each panel, the upper graphs show the predicted relative transcript abundance for ambient (black), warm (red) and cool (light blue) conditions, and the lower graphs represent the differences in transcript abundance against the ambient condition. In each graph, the solid line and the shaded region are the median and the 95% credible interval of the posterior distribution. Panels A and B include estimation of rhythmicity and peak time relative to solar dawn of model predictions, using JTK_CYCLE. Shaded boxes indicate the period between sunset and sunrise.

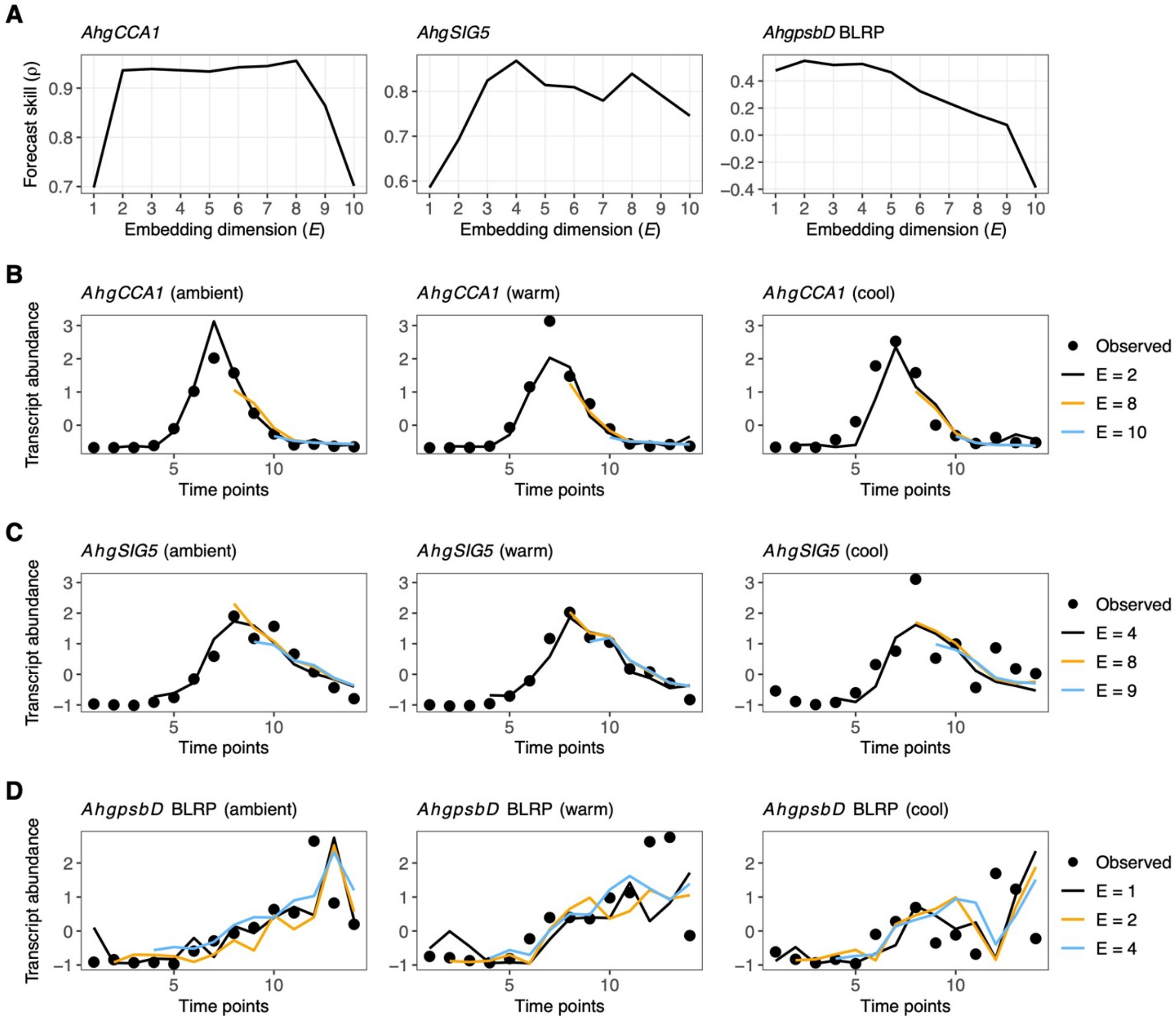


**Fig. S17.** Determination of optimal embedding dimensions of components of a circadian signalling pathway for temperature manipulation experiments in September 2016. (A) Evaluation of optimal embedding dimension *E* for each pathway component, with the forecast skill measured using ρ. A greater forecast skill was used to select the most appropriate embedding dimension. (B-D) Prediction of the fluctuations of *AhgCCA1*, *AhgSIG5* and *AhgpsbD* BLRP for several *E* values. Dots and lines are the observed (mean of replicates) and predicted values, respectively. The optimal *E* values determined were 2 for *AhgCCA1*, 4 for *AhgSIG5*, and 2 for *AhgpsbD* BLRP (see Methods for more detail).

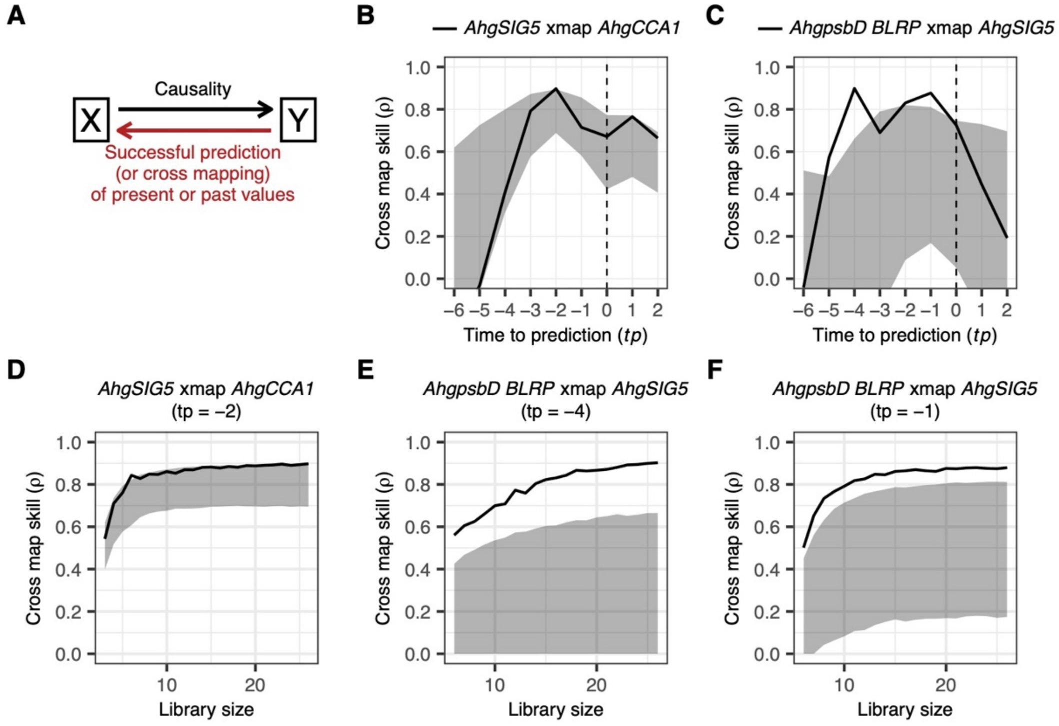


**Fig. S18.** Evaluation of causal relationships between components of a circadian signalling pathway in a natural population of *A. halleri* in September 2015. (A) The direction of causality (from *X* to *Y*) and that of prediction (cross mapping) in convergent cross mapping (CCM) (from *Y* to *X*) is opposite. (B-C) Estimation of causality between pairs of pathway components, across a range of time delays between the pathway component (time to prediction, *tp*) for data in September 2015. Cross map skill (ρ) provides a measure of the potential causality strength between the two variables. (D-F) Test of convergence, i.e., an improvement in cross map skill according to increase in a library size (number of time points used to reconstruct a state space), for each time lag with a significant cross map skill. In B-F, the solid line represents the cross map skill between pathway components, and shaded area represents the 95% interval of the cross map skill using 1,000 diel surrogate time series as the explanatory variable that reflects the same degree of oscillation but with the sequence of variation randomized (i.e., null model).

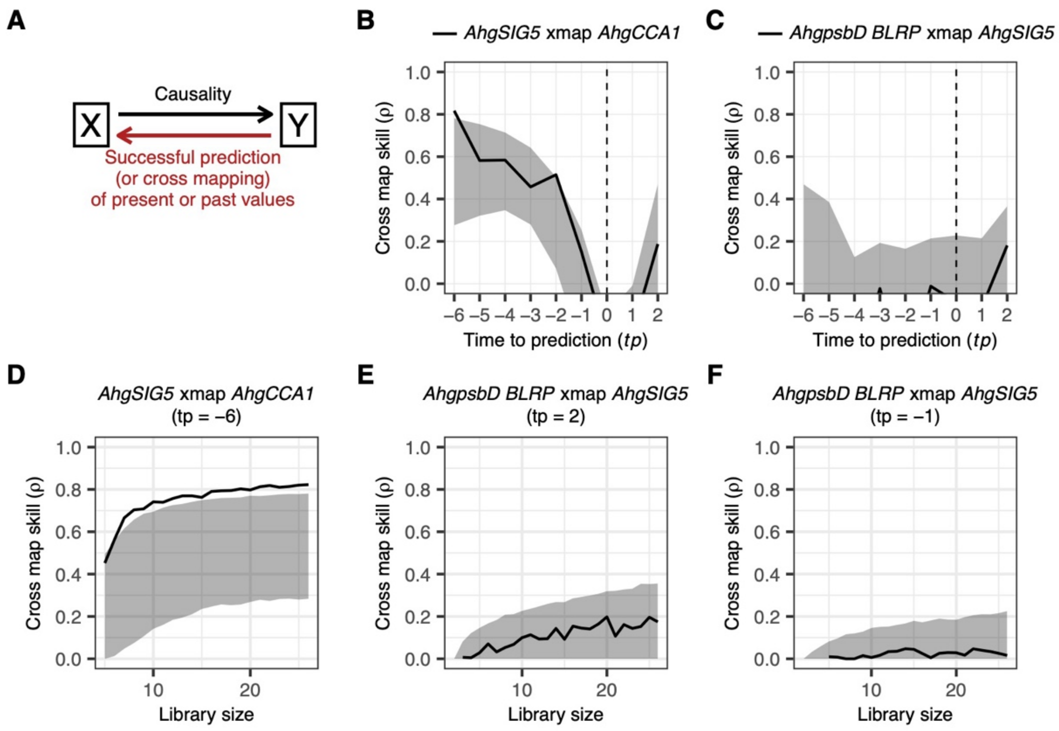


**Fig. S19.** Evaluation of causal relationships between components of a circadian signalling pathway in a natural population of *A. halleri* in March 2015. (A) The direction of causality (from *X* to *Y*) and that of prediction (cross mapping) in convergent cross mapping (CCM) (from *Y* to *X*) is opposite. (B-C) Estimation of causality between pairs of pathway components, across a range of time delays between the pathway component (time to prediction, *tp*) for data in March 2015. Cross map skill (ρ) provides a measure of the potential causality strength between the two variables. (D-F) Test of convergence, i.e., an improvement in cross map skill according to increase in a library size (number of time points used to reconstruct a state space), for each time lag with a high cross map skill. In B-F, the solid line represents the cross map skill between pathway components, and shaded area represents the 95% interval of the cross map skill using 1,000 diel surrogate time series as the explanatory variable that reflects the same degree of oscillation but with the sequence of variation randomized (i.e., null model).

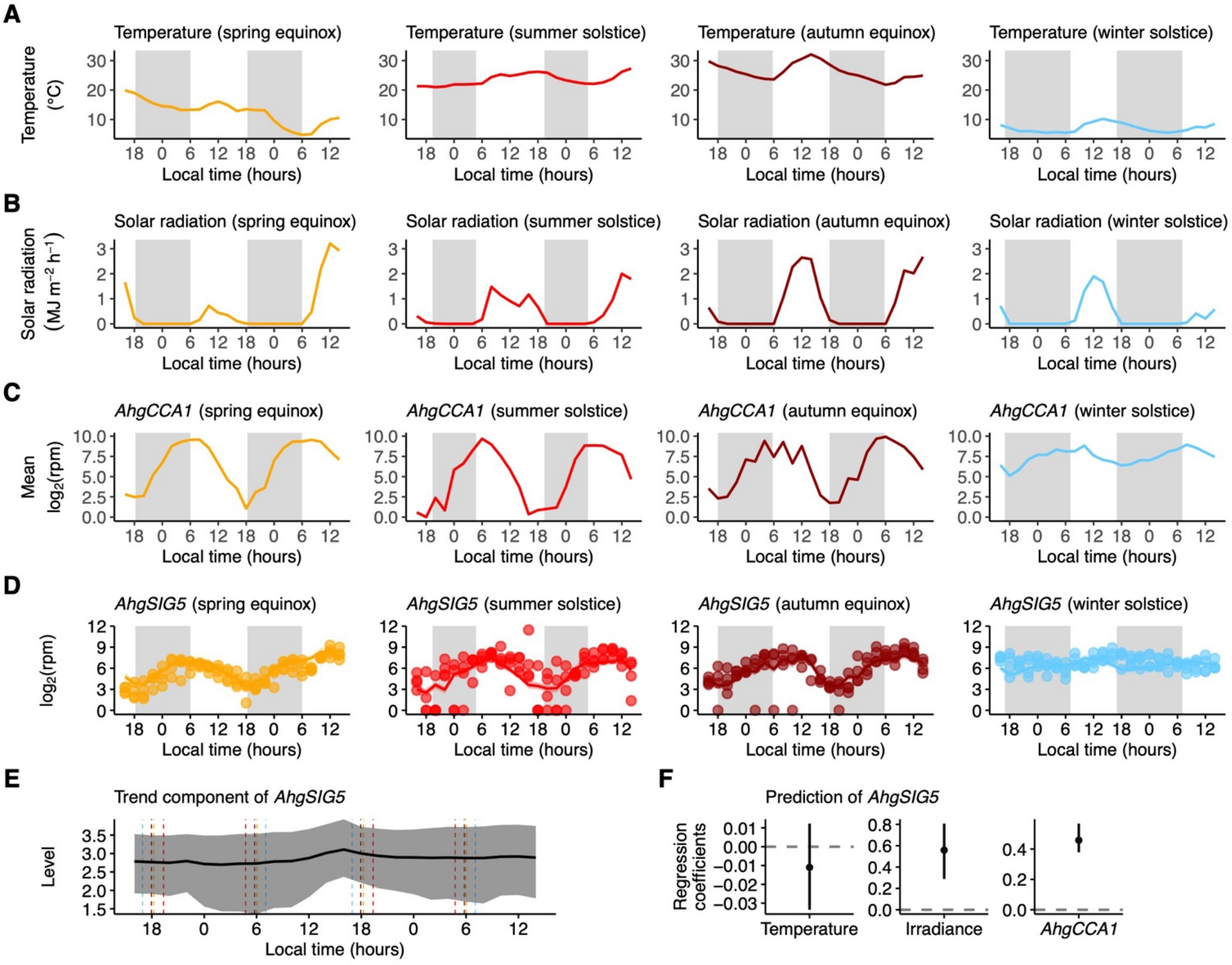


**Fig. S20.** Prediction of *AhgSIG5* signalling pathway dynamics in four seasons with different day length in a natural population of *A. halleri*. (A, B) Diel fluctuations in (A) ambient temperature and (B) solar radiation, at 2-hour intervals in the four sets of 48-h sampling period in 2013. (C) *AhgCCA1* transcript abundance averaged over four biological replicates during the sampling period. (D) Bayesian estimation of the local level model with exogenous variables (LLMX) for *AhgSIG5* transcript dynamics during the sampling period. Modelled transcript dynamics (lines and shaded area representing the median and the 95% confidence interval of the posterior distribution, respectively) are superimposed upon observed mean transcript abundance (circles). (E) Bayesian estimation of trend component of *AhgSIG5* transcript (line and shaded area representing the median and the 95% confidence interval, respectively). Vertical dashed lines indicate the times of sunrise and sunset in each season. (F) Bayesian estimation of regression coefficient of environmental variables and a potential upstream regulator (*AhgCCA1*). Dots and error bars represent the median and the 95% confidence interval, respectively. Shaded boxes on A-D indicate the period between sunset and sunrise. LLMX analysis used data from 4 replicate plants per season. Analysis used RNA-seq data from (1).

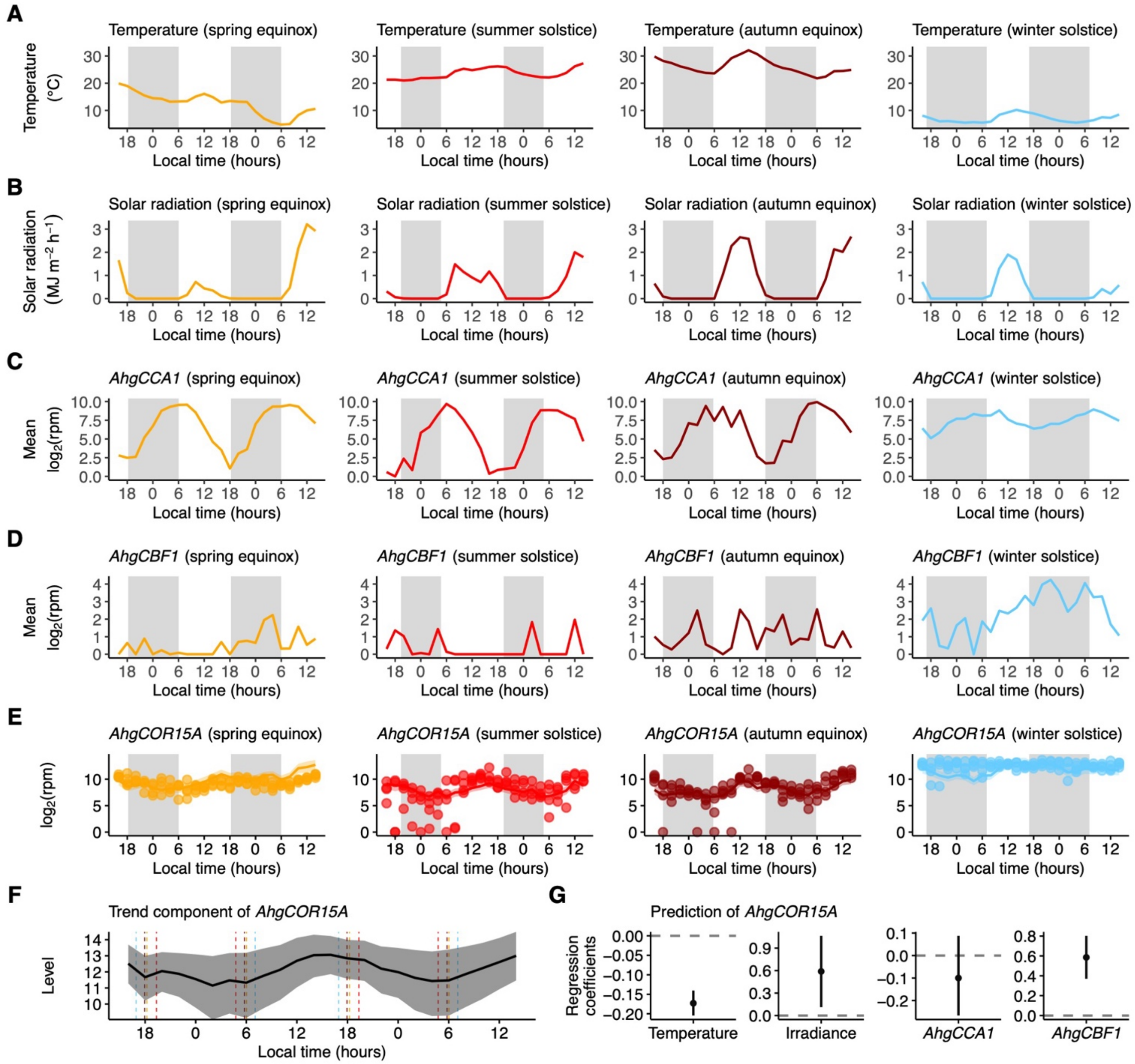


**Fig. S21.** Prediction of *AhgCOR15A* signalling pathway dynamics in four seasons with different day length in a natural population of *A. halleri*. (A, B) Diel fluctuations in (A) ambient temperature and (B) solar radiation, at 2-hour intervals in the four sets of 48-h sampling period in 2013. (C, D) (C) *AhgCCA1* and (D) *AhgCBF1* transcript abundance averaged over four biological replicates during the sampling period. (E) Bayesian estimation of the local level model with exogenous variables (LLMX) for *AhgCOR15A* transcript dynamics during the sampling period. Modelled transcript dynamics (lines and shaded area representing the median and the 95% confidence interval of the posterior distribution, respectively) are superimposed upon observed mean transcript abundance (circles). (F) Bayesian estimation of trend component of *AhgCOR15A* transcript (line and shaded area representing the median and the 95% confidence interval, respectively). Vertical dashed lines indicate the times of sunrise and sunset in each season. (G) Bayesian estimation of regression coefficient of environmental variables and potential upstream regulators (*AhgCCA1* and *AhgCBF1*). Dots and error bars represent the median and the 95% confidence interval, respectively. Shaded boxes on A-E indicate the period between sunset and

sunrise. LLMX analysis used data from 4 replicate plants per season. Analysis used RNA-seq data from (1).

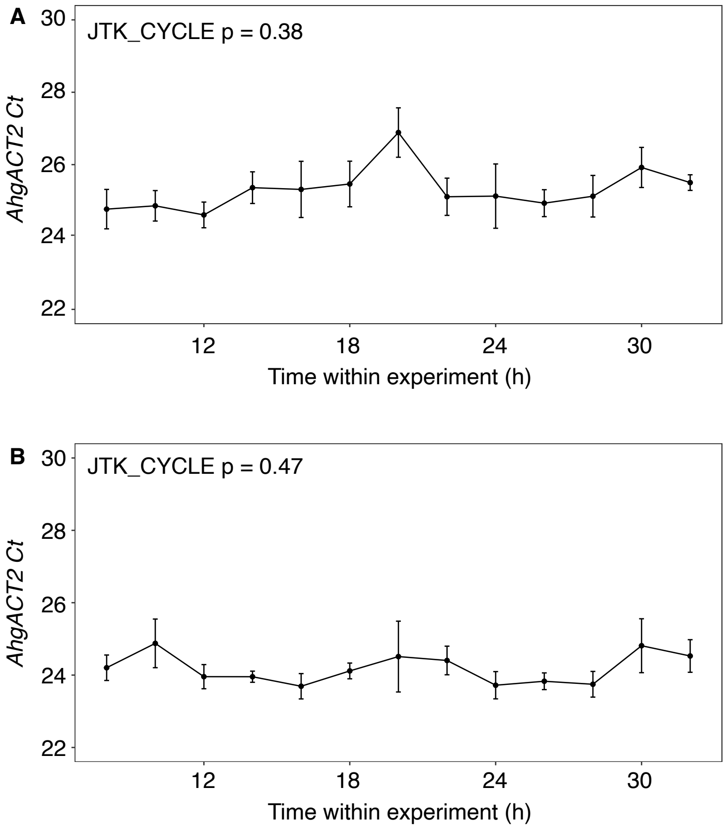


**Fig. S22.** *AhgACT2* reference transcript was not rhythmic over diel timecourses in our experiments. Ct for *AhgACT2* from RT-qPCR analysis of (A) sun and (B) shade datasets collected during September 2015. Data are mean Ct ± s.e.m; $n$ = 6. p-values provided for statistical test for significant rhythmicity, using the JTK_CYCLE algorithm (13).

**Table S1.** Primers used for RT-qPCR analysis of transcript abundance in *Arabidopsis halleri* subsp. *gemmifera*.

| **Primer** | **Sequence (5' to 3')** |
|---|---|
| *AhgACT2* (forward) | TCAGATGCCCAGAAGTGTTGTTCC |
| *AhgACT2* (reverse) | CCGTACAGATCCTTCCTGATATCC |
| *AhgpsbD* BLRP (forward) | GGAAATCCGTCGATATCTCT |
| *AhgpsbD* BLRP (reverse) | CTCTCTTTCTCTAGGCAGGAAC |
| *AhgSIG5* (forward) | GTGTTGGAGCTAATAACAGCAGACA |
| *AhgSIG5* (reverse) | TGTCGAATAACCAGACTCTCTTTCG |
| *AhgCCA1* (forward) | GCACTTTCCGCGAGTTCTTG |
| *AhgCCA1* (reverse) | TGACTCCTTTCTTATCCTGTTATTCTG |

**Table S2.** Estimated parameter values in the local level model with exogenous variables (LLMX) for *AhgSIG5*. The 2.5 %, 50.0 % and 97.5 % points of 4,000 MCMC samples obtained from posterior distributions are shown.

| **Parameter** | **2.5%** | **50.0%** | **97.5%** |
|---|---|---|---|
| μ[1] | 1.32 | 1.74 | 2.16 |
| μ[2] | 1.13 | 1.48 | 1.85 |
| μ[3] | 1.11 | 1.49 | 1.87 |
| μ[4] | 0.83 | 1.17 | 1.50 |
| μ[5] | 0.56 | 0.89 | 1.23 |
| μ[6] | 0.49 | 0.84 | 1.16 |
| μ[7] | 0.35 | 0.71 | 1.08 |
| μ[8] | -0.20 | 0.27 | 0.72 |
| μ[9] | -0.30 | 0.20 | 0.68 |
| μ[10] | 0.02 | 0.50 | 0.98 |
| μ[11] | 0.58 | 0.99 | 1.41 |
| μ[12] | 1.19 | 1.61 | 2.06 |
| μ[13] | 1.29 | 1.76 | 2.24 |
| β_temp | -0.06 | -0.05 | -0.03 |
| β_light | -1.00E-04 | 2.00E-04 | 5.00E-04 |
| β_CCA1 | 0.05 | 0.09 | 0.13 |
| σ_μ | 0.23 | 0.41 | 0.72 |
| σ_Y | 0.77 | 0.84 | 0.91 |

**Table S3.** Estimated parameter values in the local level model with exogenous variables (LLMX) for *AhgpsbD* BLRP. The 2.5 %, 50.0 % and 97.5 % points of 4,000 MCMC samples obtained from posterior distributions are shown.

| Parameter | 2.5% | 50.0% | 97.5% |
|---|---|---|---|
| μ[1] | -0.09 | 0.37 | 0.90 |
| μ[2] | 0.04 | 0.48 | 1.07 |
| μ[3] | -0.01 | 0.41 | 0.89 |
| μ[4] | 0.00 | 0.39 | 0.83 |
| μ[5] | -0.03 | 0.35 | 0.76 |
| μ[6] | -0.04 | 0.33 | 0.73 |
| μ[7] | 0.00 | 0.35 | 0.76 |
| μ[8] | -0.03 | 0.35 | 0.74 |
| μ[9] | -0.02 | 0.35 | 0.75 |
| μ[10] | 0.02 | 0.41 | 0.85 |
| μ[11] | 0.08 | 0.50 | 1.03 |
| μ[12] | 0.10 | 0.60 | 1.22 |
| μ[13] | 0.15 | 0.76 | 1.58 |
| β_temp | 0.02 | 0.04 | 0.06 |
| β_light | -5.00E-04 | -2.00E-04 | 2.00E-04 |
| β_SIG5 | 0.04 | 0.29 | 0.52 |
| σ_μ | 0.02 | 0.16 | 0.45 |
| σ_Y | 1.00 | 1.08 | 1.18 |

## SI References


1. A. J. Nagano *et al.*, Annual transcriptome dynamics in natural environments reveals plant seasonal adaptation. *Nature Plants* **5**, 74-83 (2019).
2. S. Aikawa, M. J. Kobayashi, A. Satake, K. K. Shimizu, H. Kudoh, Robust control of the seasonal expression of the Arabidopsis *FLC* gene in a fluctuating environment. *Proceedings of the National Academy of Sciences* **107**, 11632-11637 (2010).
3. H. Kudoh, M. N. Honjo, H. Nishio, J. Sugisaka, "The long-term "*in natura*" study sites of *Arabidopsis halleri* for plant transcription and epigenetic modification analyses in natural environments" in Plant Transcription Factors: Methods and Protocols, N. Yamaguchi, Ed. (Springer New York, New York, NY, 2018), 10.1007/978-1-4939-8657-6_3, pp. 41-57.
4. M. N. Honjo, H. Kudoh, *Arabidopsis halleri*: a perennial model system for studying population differentiation and local adaptation. *AoB PLANTS* **11**, article plz076 (2019).
5. R. V. Briskine *et al.*, Genome assembly and annotation of *Arabidopsis halleri*, a model for heavy metal hyperaccumulation and evolutionary ecology. *Molecular Ecology Resources* **17**, 1025-1036 (2017).
6. H. Kudoh, Molecular phenology in plants: *in natura* systems biology for the comprehensive understanding of seasonal responses under natural environments. *New Phytologist* **210**, 399-412 (2016).
7. M. Kamitani, A. J. Nagano, M. N. Honjo, H. Kudoh, RNA-Seq reveals virus–virus and virus–plant interactions in nature. *FEMS Microbiology Ecology* **92**, article fiw176 (2016).
8. P. H. Hoffer, D. A. Christopher, Structure and blue-light-responsive transcription of a chloroplast *psbD* promoter from *Arabidopsis thaliana*. *Plant Physiology* **115**, 213-222 (1997).
9. K. A. Franklin, Shade avoidance. *New Phytologist* **179**, 930-944 (2008).
10. K. J. Livak, T. D. Schmittgen, Analysis of relative gene expression data using real-rime quantitative PCR and the 2−ΔΔCT method. *Methods* **25**, 402-408 (2001).
11. F. E. Belbin *et al.*, Integration of light and circadian signals that regulate chloroplast transcription by a nuclear-encoded sigma factor. *New Phytologist* **213**, 727-738 (2017).
12. Z. B. Noordally *et al.*, Circadian control of chloroplast transcription by a nuclear-encoded timing signal. *Science* **339**, 1316-1319 (2013).
13. M. E. Hughes, J. B. Hogenesch, K. Kornacker, JTK_CYCLE: An efficient nonparametric algorithm for detecting rhythmic components in genome-scale data sets. *Journal of Biological Rhythms* **25**, 372-380 (2010).
14. A. Gelman, J. Hill, M. Yajima, Why we (usually) don't have to worry about multiple comparisons. *Journal of Research on Educational Effectiveness* **5**, 189-211 (2012).
15. A. Gelman, Prior distributions for variance parameters in hierarchical models (comment on article by Browne and Draper). *Bayesian Analysis* **1**, 515-534 (2006).

16. M. Kai, H. Yokoi, Performance evaluation of information criteria for estimating a shape parameter in a Bayesian state-space biomass dynamics model. *Fisheries Research* **219**, 105326 (2019).
17. D. J. Pascoe, A. Smyrli, T. van Doorsselaere, A. M. Broomhall, Bayesian analysis of quasi-periodic pulsations in stellar flares. *The Astrophysical Journal* **905**, article 70 (2020).
18. S. Peluso, S. Chib, A. Mira, Semiparametric multivariate and multiple change-point modeling. *Bayesian Analysis* **14**, 727-751 (2019).
19. E. Austin, G. Romano, I. A. Eckley, P. Fearnhead, Online non-parametric changepoint detection with application to monitoring operational performance of network devices. *Computational Statistics & Data Analysis* **177**, 107551 (2023).
20. F. Takens, "Dynamical systems and turbulence" in Lecture Notes in Mathematics, D. Rand, L. S. Young, Eds. (Springer-Verlag, New York, 1981), vol. 898, pp. 366-381.
21. C.-W. Chang, M. Ushio, C.-h. Hsieh, Empirical dynamic modeling for beginners. *Ecological Research* **32**, 785-796 (2017).
22. G. Sugihara, R. M. May, Nonlinear forecasting as a way of distinguishing chaos from measurement error in time series. *Nature* **344**, 734-741 (1990).
23. G. Sugihara *et al.*, Detecting causality in complex ecosystems. *Science* **338**, 496-500 (2012).
24. H. Ye, E. R. Deyle, L. J. Gilarranz, G. Sugihara, Distinguishing time-delayed causal interactions using convergent cross mapping. *Scientific Reports* **5**, 14750 (2015).
25. A. T. Clark *et al.*, Spatial convergent cross mapping to detect causal relationships from short time series. *Ecology* **96**, 1174-1181 (2015).
26. H. Nishio *et al.*, Repressive chromatin modification underpins the long-term expression trend of a perennial flowering gene in nature. *Nature Communications* **11**, article 2065 (2020).
27. M. F. Covington, J. N. Maloof, M. Straume, S. A. Kay, S. L. Harmer, Global transcriptome analysis reveals circadian regulation of key pathways in plant growth and development. *Genome Biology* **9**, R130 (2008).
28. A. N. Dodd *et al.*, The *Arabidopsis* circadian clock incorporates a cADPR-based feedback loop. *Science* **318**, 1789 (2007).
29. K. D. Edwards *et al.*, *FLOWERING LOCUS C* mediates natural variation in the high-temperature response of the *Arabidopsis* circadian clock. *The Plant Cell* **18**, 639-650 (2006).
30. S. L. Harmer *et al.*, Orchestrated transcription of key pathways in *Arabidopsis* by the circadian clock. *Science* **290**, 2110-2113 (2000).
31. T. P. Michael *et al.*, Network discovery pipeline elucidates conserved time-of-day–specific cis-regulatory modules. *PLOS Genetics* **4**, e14 (2008).
